# In situ evidence for serpentinization within the Máaz formation, Jezero crater, Mars

**DOI:** 10.1126/sciadv.adr8793

**Published:** 2025-07-02

**Authors:** Nicholas J. Tosca, Michael M. Tice, Joel A. Hurowitz, David A. K. Pedersen, Jesper Henneke, Lucia Mandon, Francis M. McCubbin, Oliver Ross, Po-Yen Tung, Richard J. Harrison, An Li, Mariek E. Schmidt, Tanya V. Kizovski, Yang Liu, Lisa Mayhew, Michael W. M. Jones, Josh Labrie, Scott Davidoff, Abigail C. Allwood, Olivier Beyssac, Adrian Brown, Morgan Cable, Jade Comellas, Benton C. Clark, Adrian E. Galvin, Briony Horgan, Christopher M. Heirwegh, Peter Nemere, Brendan J. Orenstein, Cathy Quantin-Nataf, Clément Royer, Allan Treiman, Lawrence A. Wade, Roger Wiens, Austin P. Wright

**Affiliations:** ^1^ Department of Earth Sciences, University of Cambridge, Cambridge, CB2 3EQ, UK.; ^2^ Department of Geology and Geophysics, Texas A&M University, College Station, TX 77843, USA.; ^3^ Department of Geosciences, Stony Brook University, Stony Brook, NY 11794, USA.; ^4^ Technical University of Denmark, DTU Space, Kongens Lyngby, 2800, Denmark.; ^5^ University Grenoble Alpes, CNRS, IPAG, 38000 Grenoble, France.; ^6^ ARES Division, NASA Johnson Space Center, Houston, TX 77058, USA.; ^7^ Department of Earth and Space Sciences, University of Washington, Seattle, WA 98195, USA.; ^8^ Department of Earth Sciences, Brock University, St. Catharines, ON L2S 3A1, Canada.; ^9^ Jet Propulsion Laboratory, California Institute of Technology, Pasadena, CA 91109, USA.; ^10^ Geological Sciences, University of Colorado Boulder, Boulder, CO 80309, USA.; ^11^ Central Analytical Research Facility, Queensland University of Technology, Brisbane, 4000, Australia.; ^12^ School of Chemistry and Physics, Queensland University of Technology, Brisbane, 4000, Australia.; ^13^ Centre for Planetary Surface Exploration, Queensland University of Technology, Brisbane, 4000, Australia.; ^14^ IMPMC, Sorbonne Université, 75005 Paris, France.; ^15^ Plancius Research, Severna Park, MD, 21146, USA.; ^16^ HIGP, University of Hawai’i at Manoa, Honolulu, HI 96822, USA.; ^17^ Space Science Institute, Boulder, CO 80301, USA.; ^18^ Department of Earth, Atmospheric and Planetary Sciences, Purdue University, West Lafayette, IN 47907 USA.; ^19^ Earth and Atmospheric Sciences, Queensland University of Technology, Brisbane, 4000, Australia.; ^20^ Université de Lyon, UCBL, ENSL, CNRS, LGL-TPE, Lyon, France.; ^21^ Lunar and Planetary Institute (USRA), Houston, TX 77058, USA.; ^22^ Computational Science and Engineering, Georgia Institute of Technology, Atlanta, GA 30332, USA.

## Abstract

As both a source of atmospheric H_2_ and a sink for liquid water, the serpentinization of olivine-bearing rocks is widely thought to have influenced the long-term evolution of the early martian atmosphere and hydrosphere. However, the mechanisms, timing, and global importance of this process are unconstrained, in part because the remnants of ancient serpentinizing systems have not been examined in situ. New geochemical and mineralogical data from multiple instruments aboard the Mars 2020 Perseverance rover record serpentinization and associated H_2_ production in ancient igneous rocks of the Máaz formation, exposed on the Jezero crater floor. These data, combined with petrogenetic constraints, indicate that serpentinization may have been driven by devolatilization of magmatic H_2_O, highlighting a potential link between H_2_ production and the style and tempo of magmatism within the ancient martian crust.

## Teaser

Data from the Perseverance rover record serpentinization and H_2_ production in igneous rocks exposed on the Jezero crater floor.

## INTRODUCTION

The process of serpentinization, or the aqueous alteration of olivine-bearing rocks, is thought to have shaped both habitability and the long-term planetary evolution of early Mars. Data from orbital and landed spacecraft, meteorites, and climate models indicate that the associated production of hydrous minerals and molecular hydrogen may have irreversibly depleted the global H_2_O reservoir (*1*), intermittently warmed the ancient surface (*2*, *3*), synthesised complex organic molecules (*4*), and provided an energetic substrate capable of sustaining primitive microbial life (*5*).

Yet, because serpentinizing systems have not been observed in situ, the mechanisms, spatial extent, and temporal intervals associated with this process remain poorly understood. Orbital surveys have shown that despite widespread evidence for the interaction between olivine and liquid water (*6*), Mg-rich serpentine minerals and, by inference, serpentinizing systems are rarely observed within the ancient martian crust (*7*, *8*). However, in contrast to the Mg-rich olivine found within mantle peridotite on Earth, serpentinization of Fe-rich martian olivine may instead have yielded Fe-rich serpentine and magnetite, both of which may be widespread within the ancient martian crust (*9*). Fe-rich serpentine has been identified in martian meteorites (*10*) and in sedimentary rocks at Gale crater (*11*), but a lack of geologic context for how serpentinization might have occurred has left both their timings and mechanisms unconstrained.

Here, we report in situ texturally resolved geochemical and mineralogical data from multiple instruments aboard the Mars 2020 Perseverance rover that directly record mineralogical and textural attributes consistent with products of serpentinzation, by inference its production of H_2_, and thereby constrain its timing and mechanism within basaltic rocks exposed within the Jezero crater floor. In combination with petrogenetic constraints (*12*), these data suggest that serpentinization may have been driven by devolatilization of magmatic H_2_O, raising the possibility that the timing and extent of serpentinization and associated H_2_ production on early Mars may have been controlled, at least in part, by the style and tempo of magmatism in Mars’ relatively thick basaltic crust. Because they likely record a geological process of planetary-scale importance, the samples collected from these rocks and cached by the Perseverance rover are therefore among the highest-priority targets for potential Mars sample return.

### Geological context

**Fig. 1.**
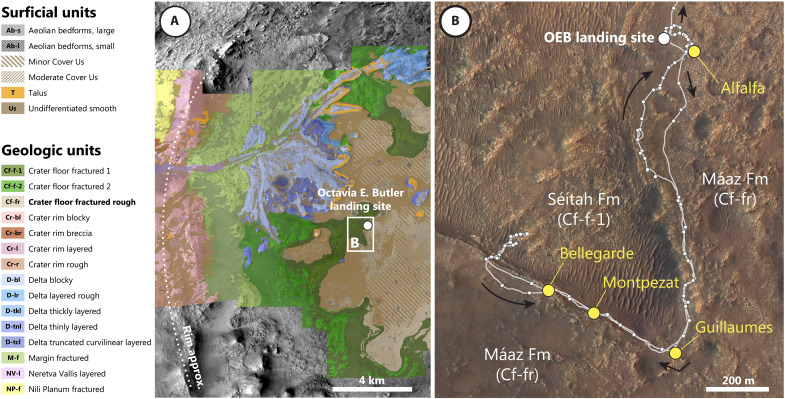
Geological context for the Máaz formation abrasion patches. (**A**) Photogeologic map of western Jezero crater [modified from (*14*)]. (**B**) Rover traverse during the first ~370 sols and location of four abraded targets of the Máaz formation, overlayed on a HiRISE mosaic (*77*). OEB, Octavia E. Butler.

The Perseverance rover landed within a spatially extensive, mineralogically, and morphologically distinct bedrock unit originally designated Cf-fr (Fig. 1) (*13*–*15*). This unit overlies an extensive fractured unit (Cf-f-1) that has been interpreted to occupy the lowest relative stratigraphic position exposed within the crater floor (*16*, *17*). On the basis of similar spectral characteristics and relative stratigraphic position, this underlying unit may be related to regional olivine-bearing units exposed across the western Isidis region (*16*, *17*). The Cf-fr unit has been dated at 2.6 ± 0.6 billion years (Ga) (*18*), although burial and resurfacing may have contributed to an artificially young crater age; an alternative interpretation places the formation of the Cf-fr unit before the main phase of delta sediment deposition to the west (i.e., >3 Ga) (*19*), a hypothesis supported by recent subsurface radar imaging of the contact between the crater floor and deltaic deposits preserved in western Jezero crater (*20*). After landing, the Cf-fr unit was renamed the Máaz formation (after the Navajo word for Mars).

The initial phase of the mission investigated Máaz formation targets along a southward traverse until the rover reached an area named the Artuby ridge, which exposes a transition from the Cf-fr unit to an underlying unit characterized by strong olivine signatures in orbital reflectance data (Cf-f-1); this unit was subsequently named the Séítah formation (“amidst the sands” in Navajo) (*15*, *21*, *22*).

The rover continued to investigate both Máaz and Séítah formation targets until returning along the initial traverse to begin investigation of the delta front deposits. Along this initial traverse, lasting approximately 370 sols, eight samples were collected; two sample pairs from the Máaz formation and two sample pairs from the Séítah formation (Fig. 1) (*23*). The acquisition of each sample was preceded by detailed in situ investigation of an abraded rock target (or “patch”) adjacent to where sampling was to be attempted. The four abraded rock targets within the Máaz formation are the focus of this study (Fig. 1). Three abraded targets were examined within the Séítah formation, the results of which have been presented in previous publications (*15*, *21*, *24*–*27*).

In the vicinity of the landing site, the Máaz formation consists of blocky, massive, and layered bedrock with rare vesicular texture (*15*, *22*, *28*–*30*). Analysis of outcrop and abraded patches by the PIXL (planetary instrument for x-ray lithochemistry), SHERLOC (scanning habitable environments with raman and luminescence for organics and chemicals), SuperCam, and Mastcam-Z instruments has classified the rocks as basaltic/trachy-basalts with intergrown pyroxene, plagioclase feldspar, minor olivine, and Fe-Ti oxides (*12*, *15*, *22*, *28*, *29*). The four abraded patches within the Máaz formation exhibit interlocking crystals indicative of igneous texture with variable heterogeneous red to brown coloration (*12*, *15*, *29*).

## RESULTS

### Geochemical, mineralogical, and textural constraints from PIXL XRF and SuperCam-IR

**Fig. 2.**
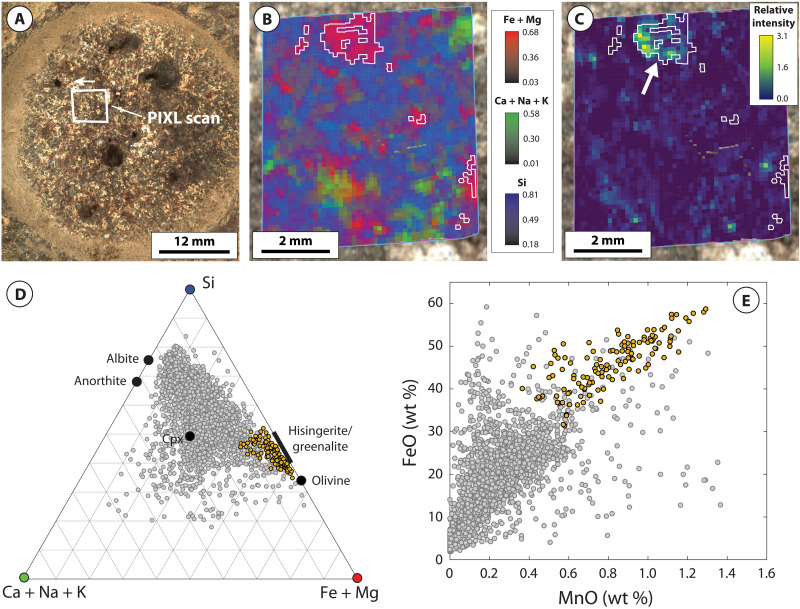
PIXL micro-XRF and back-reflected x-ray diffraction data for the Guillaumes abrasion patch. (**A**) WATSON image of the Guillaumes abrasion patch within the Máaz formation, acquired on sol 160. Arrow indicates orientation of PIXL scans in (B) and (C). (**B**) Tri-color map of the molar fraction of Fe + Mg (red), Ca + Na + K (green), and Si (blue). White outlines illustrate the location of Fe-Si material. (**C**) Relative intensity of the back-reflected x-ray diffraction peak at 6 keV, arising from fayalitic olivine. White outlines illustrate the location of Fe-Si material. White arrow indicates crystalline domains dissected by, or in intimate association with, Fe-Si material. (**D**) Ternary plot of the molar fraction of Fe + Mg [the value of which corresponds to the intensity of red color in (B)], Ca + Na + K [the value of which corresponds to the intensity of green color in (B)], and Si [the value of which corresponds to the intensity of blue color in (B)] for individual PIXL XRF spot analyses. Orange points correspond to Fe-Si material. (**E**) MnO versus FeO for individual PIXL XRF spot analyses. Orange points correspond to Fe-Si material. Gray points correspond to the remainder of PIXL XRF scan.

**Fig. 3.**
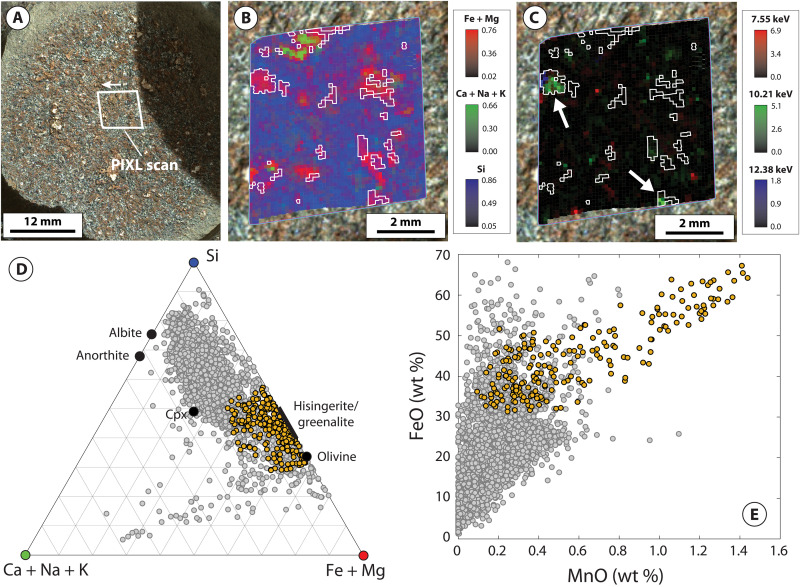
PIXL micro-XRF and back-reflected x-ray diffraction data for the Bellegarde abrasion patch. (**A**) WATSON image of the Bellegarde abrasion patch within the Máaz formation, acquired on sol 185. Arrow indicates orientation of PIXL scans in (B) and (C). (**B**) Tri-color map of the molar fraction of Fe + Mg (red), Ca + Na + K (green), and Si (blue). White outlines illustrate the location of Fe-Si material. (**C**) Relative intensity of the back-reflected x-ray diffraction peaks at 7.55, 10.21, and 12.38 keV, arising from fayalitic olivine. White outlines illustrate the location of Fe-Si material. White arrows indicate crystalline domains dissected by, or in intimate association with, Fe-Si material. (**D**) Ternary plot of the molar fraction of Fe + Mg [the value of which corresponds to the intensity of red color in (B)], Ca + Na + K [the value of which corresponds to the intensity of green color in (B)], and Si [the value of which corresponds to the intensity of blue color in (B)] for individual PIXL XRF spot analyses. Orange points correspond to Fe-Si material. (**E**) MnO versus FeO for individual PIXL XRF spot analyses. Orange points correspond to Fe-Si material. Gray points correspond to the remainder of PIXL XRF scan.

**Fig. 4.**
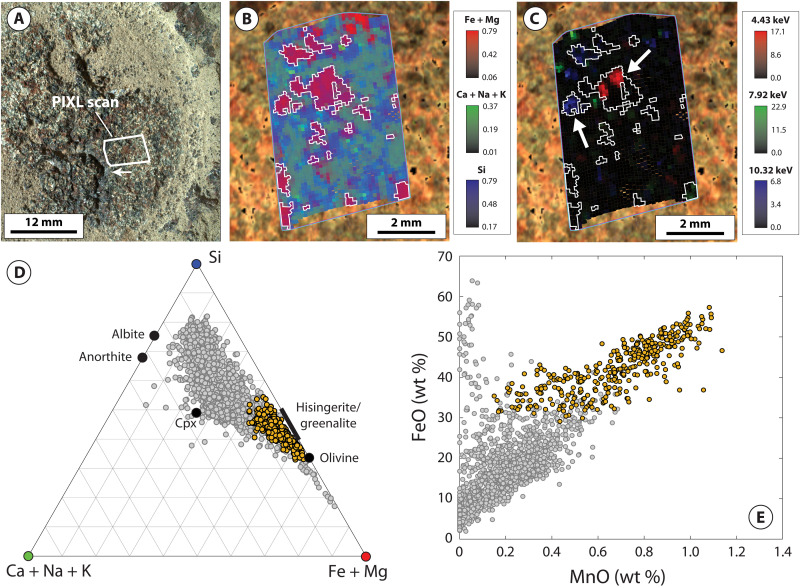
PIXL micro-XRF and back-reflected x-ray diffraction data for the Montpezat abrasion patch. (**A**) WATSON image of the Montpezat abrasion patch within the Máaz formation, acquired on sol 346. Arrow indicates orientation of PIXL scans in (B) and (C). (**B**) Tri-color map of the molar fraction of Fe + Mg (red), Ca + Na + K (green), and Si (blue). White outlines illustrate the location of Fe-Si material. (**C**) Relative intensity of the back-reflected x-ray diffraction peaks at 4.43, 7.92, and 10.32 keV, arising from fayalitic olivine. White outlines illustrate the location of Fe-Si material. White arrows indicate crystalline domains dissected by, or in intimate association with, Fe-Si material. (**D**) Ternary plot of the molar fraction of Fe + Mg [the value of which corresponds to the intensity of red color in (B)], Ca + Na + K [the value of which corresponds to the intensity of green color in (B)], and Si [the value of which corresponds to the intensity of blue color in (B)] for individual PIXL XRF spot analyses. Orange points correspond to Fe-Si material. (**E**) MnO versus FeO for individual PIXL XRF spot analyses. Orange points correspond to Fe-Si material. Gray points correspond to the remainder of PIXL XRF scan.

**Fig. 5.**
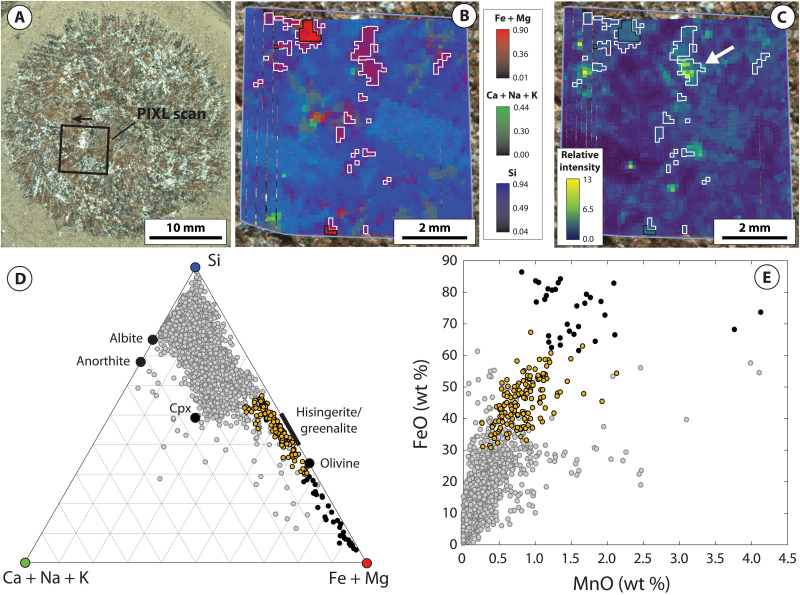
PIXL micro-XRF and back-reflected x-ray diffraction data for the Alfalfa abrasion patch. (**A**) WATSON image of the Alfalfa abrasion patch within the Máaz formation, acquired on sol 367. Arrow indicates orientation of PIXL scans in (B) and (C). (**B**) Tri-color map of the molar fraction of Fe + Mg (red), Ca + Na + K (green), and Si (blue). White outlines illustrate the location of Fe-Si material. (**C**) Relative intensity of the back-reflected x-ray diffraction peak at 5.5 keV, arising from fayalitic olivine. White outlines illustrate the location of Fe-Si material. White arrow indicates crystalline domains dissected by, or in intimate association with, Fe-Si material. (**D**) Ternary plot of the molar fraction of Fe + Mg [the value of which corresponds to the intensity of red color in (B)], Ca + Na + K [the value of which corresponds to the intensity of green color in (B)], and Si [the value of which corresponds to the intensity of blue color in (B)] for individual PIXL XRF spot analyses. Orange points correspond to Fe-Si material. Gray points correspond to the remainder of PIXL XRF scan, and black points correspond to the high FeO material dominated by magnetite. (**E**) MnO versus FeO for individual PIXL XRF spot analyses. Symbols as in (D).

PIXL x-ray fluorescence (XRF) scans acquired on the four Máaz formation abraded patches (Guillaumes, Bellegarde, Montpezat, and Alfalfa) show that, in addition to plagioclase feldspar, pyroxene, and Fe-Ti oxides, each target exhibits a distinct population of individual spot analyses dominated by Fe and Si with minor to negligible concentrations of other elements (i.e., Al and Mg; Materials and Methods and table S1). The distinct composition of these populations corresponds closely to Fe-silicate minerals characterized by a relatively high Fe/Si ratio such as Fe-rich olivine or Fe-rich 1:1 layer silicates (i.e., greenalite-hisingerite) but with apparent contamination by other minerals (Figs. 2 to 5). To isolate dominant minerals contributing to these regions, the populations were refined by excluding spot analyses contaminated by Fe-Ti oxide and/or sulfate- or chloride-bearing minerals (Materials and Methods).

The resulting populations (hereafter referred to “Fe-Si material”) share a common set of characteristics across all four abraded targets. First, in each target, the Fe-Si material corresponds to spatially discrete regions of PIXL scans ~0.5 to 2 mm in size (Figs. 2 to 5). These regions are not spatially correlated with Fe-Ti oxide minerals, which are interpreted to be of igneous origin (figs. S2 and S4) (*12*). The Fe-Si regions generally show limited spatial correlation with regions where concentrations of SO_3_ are elevated. A notable exception to this is the target Bellegarde, where the Fe-Si material surrounds SO_3_-rich void fill (figs. S1 and S3).

The Fe-Si material is also characterized by distinctly Mn-rich compositions, consistently reaching as high as 1.4 to 1.5 wt % MnO across all four targets (Figs. 2E, 3E, 4E, and 5E). In Montpezat and Alfalfa, the Fe-Si material is spatially correlated with increased counts of backscattered x-rays from the Rh-L line relative to surrounding areas (fig. S5). Because the efficiency of x-ray backscattering is inversely proportional to average atomic mass (*Z*) (*31*), this suggests that, in some scans, the Fe-Si material is associated with increased concentration of low-*Z* elements (e.g., H, O, and/or C; Materials and Methods).

Back-reflected x-ray diffraction further constrains the genetic relationships between minerals contributing to PIXL scans. These data show that large coherent scattering domains (~0.5 mm) arising from crystalline fayalitic olivine are intimately mixed with the Fe-Si material at a fine spatial scale (Figs. 2 to 5). For example, all four targets exhibit rugose and/or dissected fayalitic grain boundaries and show that the Fe-Si material does not exhibit x-ray diffraction, despite PIXL’s high probability of detecting diffraction from crystalline materials with sufficiently large coherent scattering domain size (Materials and Methods). The absence of diffraction constrains the coherent scattering domain size to less than ~45 μm (*24*), in turn representing fine or poor crystallinity. Notably, in the Montpezat abraded patch, the diffraction peak intensity at 4.43 keV, arising from a region rich in fayalitic olivine, delineates two coherent scattering domains that are in the same orientation (Fig. 4C). These domains are dissected and surrounded by a nondiffracting Fe-Si material, in turn indicating that a single crystal of fayalitic olivine was partially replaced by the Fe-Si material.

**Fig. 6.**
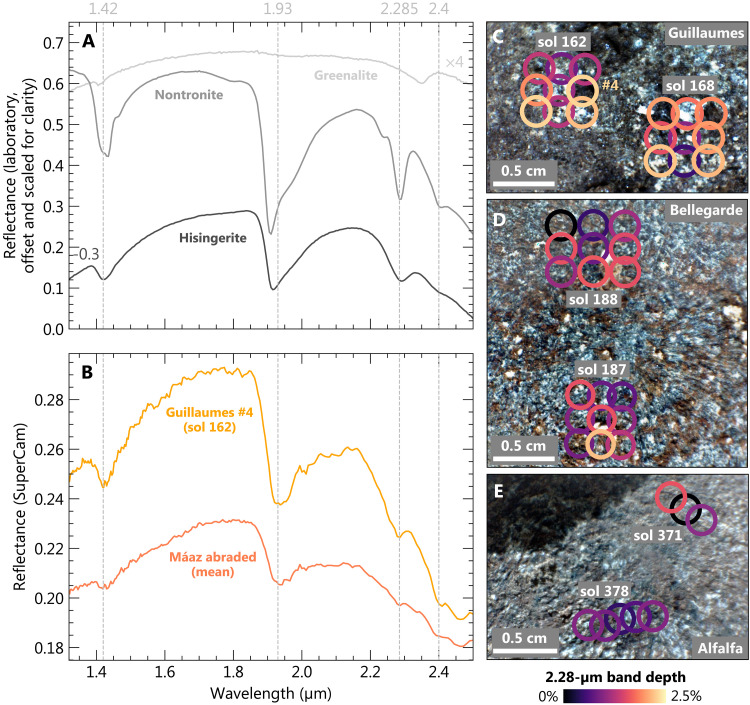
SuperCam-IR constraints on layer silicate mineralogy in Máaz formation abrasion patches. (**A**) Laboratory IR reflectance spectra obtained from greenalite, hisingerite, and Fe(III)-smectite (nontronite). Spectra are offset for clarity. (**B**) SuperCam-IR reflectance measurements obtained from the Guillaumes abraded target on sol 162 and the mean of all reflectance measurements obtained from Máaz formation abraded patches. (**C** to **E**) Locations of SuperCam-IR reflectance measurements overlain on WATSON images of the Guillaumes, Bellegarde, and Alfalfa abraded patches, respectively, with the color indicating the band depth of the 2.28-μm feature, a qualitative indication of the abundance of structural Fe(III)-OH in phyllosilicate structures.

Despite a distinctive composition and evidence for relatively high concentrations of low-*Z* elements within the Fe-Si material, PIXL alone cannot uniquely constrain its mineralogy. However, data acquired by the SuperCam–infrared (IR) instrument provide additional mineralogical constraints. SuperCam-IR data for the Máaz formation abraded patches show a spectral feature at 1.9 μm, which arises from H_2_O, in addition to distinctive 2.28- and 2.4-μm features arising from octahedrally coordinated Fe(III)-OH and Fe(III)/Mg-OH in phyllosilicate structures, respectively (*22*, *32*); Al-OH absorptions are not a dominant feature. The strength of the 2.28-μm Fe(III)-OH feature is variable across the abraded targets but is most intense in discrete regions or clusters (Fig. 6). This spectral feature occurs in both nontronite (a ferric iron-rich smectite) and hisingerite (a ferric iron-rich 1:1 layer silicate), and distinction between the two is not possible from SuperCam-IR data alone (Fig. 6) (*32*). However, PIXL scans from all four abraded targets show that the Fe-Si material exhibits an average Fe/Si ratio that is most consistent with mixtures dominated by hisingerite rather than nontronite (Materials and Methods and figs. S6 and S8).

### Mineralogical constraints from visible/near-IR reflectance

Together, constraints from PIXL XRF and SuperCam-IR data indicate that the Fe-Si material includes contributions from olivine and a secondary Fe(III)-layer silicate component which is dominated by hisingerite and/or nontronite. The visible (vis)/near-IR (NIR) reflectance properties of the Fe-Si material exposed in abraded patches provide further mineralogical constraints; these data have been acquired by both Mastcam-Z and PIXL’s micro-context camera (MCC). Horgan *et al.* (*29*) showed that some Mastcam-Z reflectance spectra acquired on the abraded patches exhibited a strong peak at ~750 nm, corresponding to hematite and/or goethite and ferrihydrite. They showed that the strongest peaks were found in the Alfalfa target, associated with visible red staining, and showed that this spectral feature becomes weaker in Bellegarde and is absent in the Montpezat abraded patch (*29*). However, Mastcam-Z cannot resolve features smaller than ~0.5 mm (*29*), which is a scale several times larger than the effective resolution of both PIXL’s MCC and XRF, and also exceeds the scale at which the Fe-Si material is spatially distributed.

MCC reflectance data provide a complement to Mastcam-Z data but with higher spatial resolution. Like Mastcam-Z, the MCC instrument is highly sensitive to the presence of Fe(III) oxide chromophore minerals. To assess and verify this sensitivity, we examined the MCC reflectance response from dust-rich versus dust-poor surfaces of the same target and compared this to PIXL XRF data. Dusty regions, which contain minor concentrations of Fe(III) oxides as constrained by the PIXL’s XRF instrument, exhibit NIR/ultraviolet (UV) ratios of up to 10 in MCC data (fig. S14, table S2, and Materials and Methods).

**Fig. 7.**
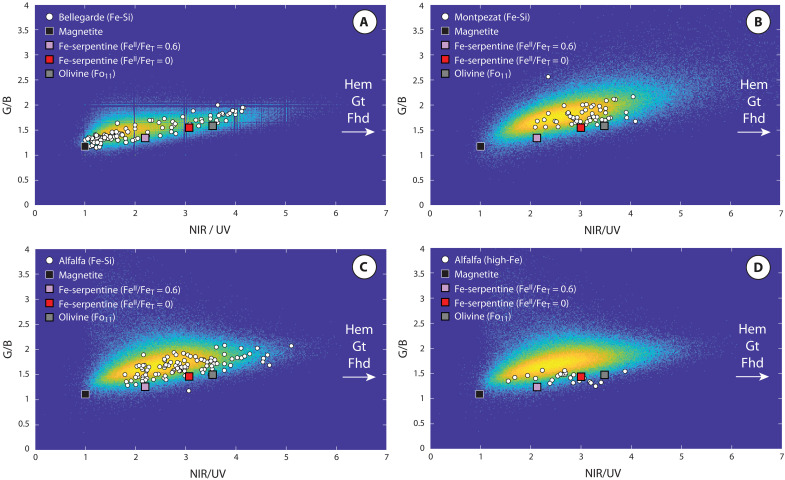
Heatmap of the NIR/UV and green/blue (G/B) absolute reflectance ratios measured by PIXL’s MCC monochomatic channels on Máaz formation abraded targets. Reflectance ratios for mineral standards are shown in squares (Materials and Methods). White points correspond to reflectance measurements for colocated PIXL XRF analyses of Fe-Si material. Hem, hematite; Gt, goethite; Fhd, ferrihydrite. (**A**) Bellegarde abrasion patch. (**B**) Montpezat abrasion patch. (**C**) Alfalfa abrasion patch Fe-Si material. (**D**) Alfalfa abrasion patch high-Fe material.

MCC reflectance data acquired on the Máaz formation abraded targets are generally consistent with results from Mastcam-Z, with some notable exceptions in regions that correlate with the Fe-Si material. In particular, MCC reflectance data show that, with the exception of a small number of points in the Alfalfa scan (Fig. 7, figs. S11 to S13, and Materials and Methods), XRF spot analyses corresponding to the Fe-Si material are not associated with the highest NIR/UV ratios measured by the MCC. In general, MCC data show that the Fe-Si material is largely characterized by moderate to low NIR/UV ratios (Fig. 7, figs. S10 to S13, and Materials and Methods). The spectral characteristics of the Fe-Si material are consistent with mineral mixtures dominated by ferroan olivine, magnetite, and/or Fe-rich 1:1 layer silicates (i.e., greenalite-hisingerite). Specifically, the moderate green/blue ratios and low NIR/UV ratios, observed across all three MCC datasets (Fig. 7, figs. S10 to S13, and Materials and Methods), may reflect a dominant contribution from Fe-rich greenalite/hisingerite which themselves range from dark green to red/brown in color depending on the relative ratio of structural Fe(II) to Fe(III) (fig. S16). Together, the comparison of Mastcam-Z data and PIXL’s MCC data indicates that Fe(III) oxide minerals are likely present in some regions of the abraded patches, with the highest concentrations in Alfalfa, but these reside in regions which are spatially distinct from Fe-Si material. The PIXL MCC data thus limit the proportion of chromophore minerals contributing to the measured composition of Fe-Si materials to relatively small concentrations. This is because vis/NIR spectra of mineral mixtures respond in a nonlinear fashion to small concentrations of chromophore minerals such as hematite, goethite, and/or ferrihydrite, particularly when they are nanocrystalline (*33*, *34*). Laboratory reflectance studies have shown that less than 1 wt % Fe(III) oxide addition to a clay mineral substrate can result in high NIR/UV ratios of the mixture as a whole (*33*, *34*). Thus, together, vis/NIR reflectance properties of the Fe-Si material are consistent with the interpretation that it is largely composed of mixtures between olivine, hisingerite, and/or nontronite.

### Geochemical, mineralogical, and textural evidence for secondary magnetite

PIXL XRF data acquired from the Alfalfa target also show a distinct population of spot analyses characterized by high Fe concentration exceeding that of the Fe-Si material (Fig. 5 and table S1). This high-Fe population is spatially associated with the Fe-Si material, with some individual spot analyses reflecting mixtures between high-Fe end-member minerals and the Fe-Si material. The high-Fe population is distinctly Ti poor and Mn rich (Fig. 5 and fig. S2); at the highest FeO concentrations, compositions correspond almost exactly to stoichiometric magnetite. However, in contrast to Fe-Ti oxides of likely igneous origin, this high-Fe population does not exhibit x-ray diffraction, constraining coherent scattering domain size to less than 45 μm (Fig. 5). Reflectance data obtained from PIXL’s MCC suggest that these same regions exhibit reflectance properties consistent with mixtures dominated by magnetite as opposed to Fe(III) oxide minerals such as hematite, goethite, and/or ferrihydrite. Because the MCC acquires reflectance measurements across four monochromatic channels (UV at 385 nm; blue at 450 nm; green at 520 nm; NIR at 720 nm), the reflectance ratio between the NIR and UV channels provides a semiquantitative constraint on the relative abundance of Fe(III) oxide chromophore minerals (which all exhibit high NIR/UV ratios) relative to fine-grained magnetite (i.e., nondiffracting, which exhibits a low NIR/UV ratio) (Fig. 7, fig. S15, and Materials and Methods). MCC data acquired on pixels that correspond to XRF spot analyses of high-Fe material yield distinctly low NIR/UV and green/blue ratios, consistent with the interpretation that magnetite is the dominant material controlling both compositional and spectral properties within these regions (Fig. 7).

The MCC reflectance properties of Fe-Si material in Bellegarde and Montpezat (no MCC measurements were acquired on the Guillaumes target) suggest that these targets could also include mixtures between Fe-Si material and phases that exhibit low NIR/UV and green/blue ratios, particularly magnetite. However, analysis of PIXL XRF data corresponding to these locations did not reveal points displaying substantially enriched FeO concentrations that might indicate substantial proportions of magnetite, as in Alfalfa; this indicates that either magnetite is not present in these regions or that mixing between finely crystalline magnetite and other phases (e.g., olivine and/or Fe-rich 1:1 layer silicates) within the Fe-Si material may be occurring at a scale smaller than the x-ray beam diameter. To examine the possibility that finely crystalline magnetite may be intimately mixed with Fe-Si material and therefore contributing to its overall compositional and spectral characteristics, we acquired micro-XRF data on polished slabs of serpentinized ferroan troctolite sampled from the 1.1 Ga Duluth Complex, USA (Materials and Methods and figs. S17 and S18). μ-XRF data acquired on these samples display a compositional range that closely corresponds to that present in all abraded targets within the Máaz formation (fig. S18), and several individual spot analyses exhibit compositions closely corresponding to magnetite. However, these laboratory analyses were acquired at higher spatial resolution compared to that of the PIXL XRF scans. When the spatial resolution of these laboratory data is artificially downgraded to match the resolution of PIXL scans, the effects of mixing between fine-grained magnetite and Fe-rich 1:1 layer silicates result in compositions corresponding more closely to olivine-greenalite/hisingerite mixtures with fewer XRF spot analyses indicative of clear mixing between end-member magnetite (as observed for Alfalfa; Fig. 5). Although the spatial resolution of PIXL XRF data does not allow for definitive identification of mixing between Fe-rich 1:1 layer silicates and fine-grained magnetite, these laboratory analyses, combined with MCC reflectance properties dominated by low NIR/UV ratio material (Fig. 7), suggest that minor concentrations of finely crystalline magnetite may also occur within Máaz formation targets.

### Mineralogical constraints from clustering and Gaussian mixture modeling

To examine whether the subpixel mixing of other end-member components present in PIXL XRF scans could have produced compositions identical to the Fe-Si material, we used a modified version of SIGMA (Spectral Interpretation using Gaussian Mixtures and Autoencoder), a machine learning approach to automatically identifying unknown chemical components and unmix their chemical signals (*35*). SIGMA takes as input the high-dimensional dataset of elemental abundances for each pixel in the XRF scan and projects it onto a two-dimensional (2D) “latent space” in a way that preserves the global structure of the data (i.e., maintains the relative positions of compositionally distinct clusters). Clustering is performed on the 2D representation using MeanShift, a nonparametric algorithm that finds clusters by iteratively shifting data points toward the densest regions in the feature space (*35*). Once chemically similar pixels are grouped into clusters, the elemental concentration data for each cluster are summed, which provides an average chemical composition of each cluster. Further description of parameter choices for these different steps is given in Materials and Methods.

Application of the SIGMA workflow to PIXL micro-XRF maps acquired on the four Máaz formation targets resulted in the detection of clusters which correspond closely to the Fe-Si material identified through manual analysis (figs. S19 to S26). For example, fig. S19 shows the 2D latent space reconstruction of the Guillaumes PIXL micro-XRF scan, including five distinct clusters. Clusters 0, 1, and 2 are characterized by distinct chemical signals, with cluster 2 representing a signal that is intermediate between 0 and 1. On the other hand, clusters 3 and 4 are significantly separated from other points in the latent space and correspond to distinct chemical end-members compared to the remaining points in the scan. These clusters are characterized by compositions that correspond to the Ca-S-Na-Cl–rich material (cluster 3) and the Fe-Si–rich material (cluster 4). The average composition and spatial distribution of cluster 4 correspond closely to that determined through manual analysis described above. Similarly, the latent space reconstruction for the Bellegard PIXL micro-XRF scan also shows two clusters with chemical signals distinct from the remainder of the scan (fig. S22): cluster 3, which exhibits an average composition and spatial distribution closely corresponding to the Fe-Si material identified manually, and cluster 4, which is rich in Ca and S and contains some points that mix with points from cluster 3. The latent space reconstruction for the Montpezat PIXL micro-XRF scan shows three clusters with chemical signals distinct from other points within the scan (fig. S24). One of these clusters (cluster 5) is dominated by Fe and Ti, corresponding closely to Fe-Ti oxide minerals identified through manual analysis and interpreted as igneous in origin. The remaining two clusters, 3 and 4, again closely correspond spatially and compositionally to the Fe-Si material identified through manual analysis. The latent space shows that these two clusters are closely related to each other, with only minor compositional differences. For example, cluster 4 is characterized by a higher Fe/Si ratio, and given its spatial distribution relative to back-reflected diffraction data (Fig. 4), we interpret this as a more olivine-rich cluster in comparison to cluster 3 which has a lower Fe/Si ratio and generally rims cluster 3. Last, the latent space reconstruction for the Alfalfa PIXL scan highlights clusters with distinct chemical signals (fig. S25). Of these, cluster 4 is separated from the rest of the data and corresponds both spatially and compositionally to the Fe-Si material identified manually. This cluster also contains a small number of data points at its most negative values in the latent *x* and *y* dimensions, which correspond to the “high-Fe” material identified as Cr- and Ti-free magnetite through manual analysis.

Together, clustering and Gaussian mixture modeling shows that in all four Máaz formation targets, the Fe-Si material is compositionally and spatially distinct from the remaining points within each scan. These results also show that the composition of the Fe-Si material is unlikely to be a result of the mixing between end-members that are present in other regions of the scans. Put differently, the analysis indicates that the Fe-Si material is its own chemical end-member and distinct in each scan.

To summarize, PIXL micro-XRF, back-reflected x-ray diffraction, SuperCam-IR, vis/NIR reflectance, and clustering and Gaussian mixture modeling place strong constraints on the mineralogical composition of the Fe-Si material. These indicate that magnetite, fayalitic olivine, hisingerite, and/or nontronite dominate the Fe-Si material in variable proportions across the four Máaz formation targets. An important question is the degree to which these data (particularly XRF points lacking diffraction, which largely exclude primary olivine) can be used to distinguish between nontronite- and hisingerite-dominant assemblages. As discussed above, these two minerals exhibit highly similar IR reflectance spectra (Fig. 6), and although nontronite is typically characterized by higher Al content and lower Fe/Si ratios relative to hisingerite, these properties are not diagnostic; Al-free nontronites have been reported in the literature, and Si-poor nontronites overlap compositionally with Si-rich hisingerites (fig. S8). Despite these similarities, one observation that more clearly distinguishes the two minerals is Mn concentration. Available chemical analyses on nontronites and hisingerites formed across a wide range of temperature and redox conditions, for which nearly monomineralic composition has been verified, show that hisingerite contains, on average, close to two orders of magnitude more Mn than nontronite (fig. S7). The reasons for this are likely to arise from crystal chemical constraints. In octahedral coordination, ionic radii for principal cations are 0.535 Å (Al^3+^), 0.645 Å (Fe^3+^), 0.72 Å (Mg^2+^), 0.78 Å (Fe^2+^), and 0.83 Å (Mn^2+^). In the nontronite structure, increases in the dimensions of the octahedral sheet (through incorporation of cations larger than Fe^3+^) result in a concomitant increase in the dimensions of tetrahedra (*36*), and the particularly large ionic radius of Mn^2+^ may prohibit the accommodation of substantial amounts of Mn^2+^ due to associated strain induced within either the octahedral and/or tetrahedral sheets. On the other hand, Fe-rich 1:1 layer silicates such as greenalite are characterized by a substantial misfit between the octahedral and tetrahedral sheets, which results from substantial concentrations of Fe^2+^ and Mn^2+^; this is alleviated through structural modulations which preserve octahedral sheet continuity and the Fe^2+^- and Mn^2+^-rich composition (*37*). For Fe^2+^- and Mn^2+^-rich hisingeritic compositions, variations in octahedral sheet structure and composition may additionally involve mechanisms such as ferri-Tschermak substitution between octahedral and tetrahedral sites, deprotonation, and/or the introduction of octahedral site vacancies (*38*). In light of these considerations, the distinctly Mn-rich composition of PIXL XRF spot analyses lacking x-ray diffraction (Figs. 2 to 5 and fig. S9), in combination with SuperCam-IR and vis/NIR reflectance data, strongly supports Fe-Si assemblages dominated by hisingerite but allow for additional contributions from nontronite and/or magnetite.

## DISCUSSION

### Temperature, timing, and mechanism of serpentinization within the Máaz formation

Together, constraints from multiple instruments aboard the Mars 2020 Perseverance rover document fayalitic olivine in close spatial association with poorly/finely crystalline alteration products. The chemical composition and UV/vis/IR spectral properties of these materials are consistent with mixtures dominated by Fe-rich 1:1 layer silicates (greenalite-hisingerite). In the Alfalfa target, these alteration products are intimately associated with finely crystalline Mn-rich/Ti- and Cr-poor magnetite. Together, this evidence reflects a style of aqueous alteration consistent with serpentinization of ferroan olivine and places constraints on the temperature, relative timing, and mechanism through which this process occurred.

The observation of both magnetite and greenalite/hisingerite-dominated assemblages derived from fayalitic olivine constrains minimum temperatures associated with serpentinization. The lower temperature stability limit of olivine in the presence of an aqueous fluid decreases with increasing Fe content, such that fayalitic olivine with the composition identified within the Máaz formation (i.e., Fo_3–20_) becomes unstable relative to secondary phases at ~150° to 200°C (*9*, *39*, *40*). In addition, experiments, thermodynamic reaction path models, and observational constraints from terrestrial serpentinizing systems show that serpentinization of Fe-rich olivine is associated with the production of greenalite/hisingerite-magnetite assemblages only at the higher end of the temperature range over which serpentinization is possible (*9*, *39*–*42*); for fayalitic olivine, magnetite is not part of the stable alteration assemblage below temperatures of ~90° to 110°C (*39*, *40*, *42*).

In situ analyses also show that while Máaz formation targets record multiple episodes of aqueous alteration (*15*, *22*, *32*), serpentinization was likely among the earliest processes to have altered these rocks. Similar to Séítah formation targets, Máaz formation abraded targets are variably and locally enriched in S and Cl, reflecting the emplacement of sulfate- and/or chlorine-bearing minerals, particularly Ca- and Mg-sulfate, and in Guillaumes specifically, Na-perchlorate (*15*, *22*, *24*). PIXL XRF maps show that Bellegarde and, to a lesser extent, the Guillaumes target display spatial correlation between sulfate minerals and Fe-rich 1:1 layer silicates, with sulfate minerals infilling voids that contain, and are rimmed by, greenalite/hisingerite (figs. S1 and S3). Together, this indicates that sulfate mineral emplacement largely postdated serpentinization. Greenalite/hisingerite-rich regions of altered olivine are likely to have provided physically incompetent, high surface area material, and pore space that hosted later-stage aqueous alteration (*15*). The composition and distribution of the S-bearing material in the Máaz formation are similar to observations acquired from abraded patches of the Séítah formation (*24*), although the extent of saline mineral emplacement differs between the two formations (*27*).

In contrast to SO_3_, Cl exhibits a markedly different distribution within Máaz formation abraded targets, suggesting that it was emplaced through a different process. PIXL XRF data show that in all targets, greenalite/hisingerite-dominated regions are also characterized by distinctly high Cl concentrations reaching up to a few weight % Cl [figs. S28 and S27 and table S1; note that the Guillaumes target preserves extreme local Cl enrichments due to a Na-perchlorate phase; (*15*)]. The strong spatial correlation between greenalite/hisingerite-rich regions and Cl suggests a genetic link between the two. We found no statistically significant correlations between Cl and any other element in these regions, and there are very low concentrations of other elements relative to Fe and Si (table S1). This suggests that Cl is either hosted in a discrete Fe-bearing phase, present as a structural substitution within Fe-rich 1:1 layer silicates, adsorbed on to their surfaces, or a combination of these factors. Relatively high Cl concentrations have been documented in serpentinized peridotites on Earth, with some reaching ~1.7 wt % (*43*), and several studies have also reported discrete Fe-Cl phases in hydrothermally altered rocks (*44*–*46*), including in Fe-rich serpentinites of the Duluth Complex, USA (*45*) and hydrothermally altered ultramafic rocks of the Bushveld Complex of South Africa (*46*). These materials exhibit red-brown coloration upon exposure to air (typically converting to akaganeite), which may result from oxidation of ferrous iron bound in discrete layered ferrous hydroxychloride phases (*46*) or double-layered hydroxides such as pyroaurite or iowaite (*47*).

Together, in contrast to the Séítah formation, Máaz formation abraded patches record strong differences in the style of olivine alteration. For example, Séítah formation targets are characterized by intergranular precipitation of carbonate and poorly crystalline silicate with minimal replacement of olivine (*24*, *26*, *48*). In contrast, Máaz formation olivine is selectively replaced by secondary products and characterized by partial to complete replacement, including dissection of individual olivine crystals likely resulting from microfracture-hosted alteration (Figs. 2 to 5). These differences suggest that although both formations record late-stage emplacement of sulfate minerals, serpentinization of Máaz formation rocks was likely mediated by fluids distinct in composition and in origin to those that altered Séítah formation olivine.

At least two processes could have contributed fluids that mediated serpentinization within the Máaz formation. First, an external aqueous fluid may have been introduced during or after the solidus was reached as Máaz formation rocks crystallized. Terrestrial serpentinizing systems mediated by external fluids are generally associated with fracture and vein networks that occur across several observational scales, which preserve evidence for multiple episodes of alteration (*49*). The Máaz formation is heavily fractured, although Perseverance has observed no evidence to date for mineralization associated with those fractures, which might be taken to reflect external fluid-mediated serpentinization involving substantial quantities of fluid (*49*). However, although external fluids cannot be ruled out on the basis of the size and scale of fractures identified to date, an important observation relates to the stratigraphic juxtaposition between the Máaz formation and the Séítah formation (*15*, *22*, *24*). Given the distinct styles of olivine alteration between these two units and their close spatial and stratigraphic proximity, serpentinization of the Máaz formation is instead more likely to have been linked to an inherent property of that unit and/or the conditions under which it was emplaced.

To that end, a second process that may have mediated serpentinization within the Máaz formation involves the release of magmatic volatiles during or after crystallization. Using chemical and textural evidence derived largely from the PIXL instrument on the four abraded targets analyzed here, Schmidt *et al.* (*12*) documented a detailed petrogenetic history for Máaz formation rocks. In particular, Schmidt *et al.* (*12*) showed that large feldspar laths preserved in the Alfalfa target were likely in thermal and chemical disequilibrium with the parent melt, indicating that the melt was staged within the crust preceding eruption. Schmidt *et al.* (*12*) also showed that all four Máaz formation targets reflect a suite of highly differentiated rocks produced through high-degree fractional crystallization and perhaps assimilation of an Fe-rich contaminant.

**Fig. 8.**
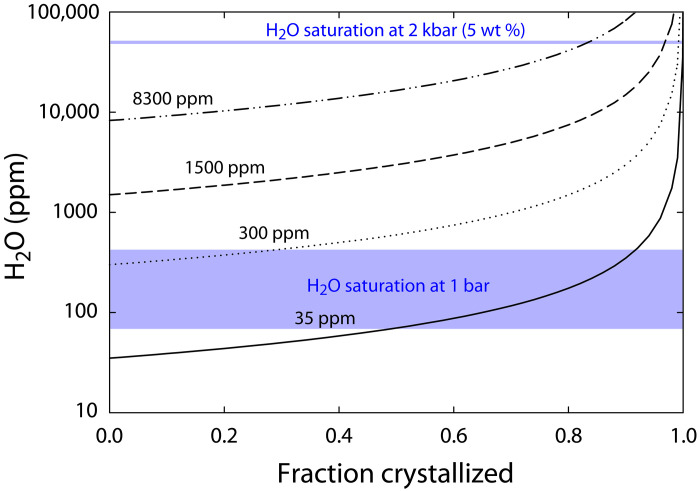
Enrichment of H_2_O abundances in silicate melt during crystallization of nominally anhydrous phases. Initial water contents of 35, 300, 1500, and 8300 parts per million (ppm) H_2_O are based on the range of parental melt H_2_O abundances that could arise from partial melting (i.e., 3 to 40% partial melting of the mantle source) of a martian mantle source based on martian mantle H_2_O abundances estimated from martian meteorite studies [i.e., 14 to 250 ppm H_2_O; (*78*)]. The saturation value of H_2_O in silicate melts is provided for 2 kbar (*50*) and 1 bar [shaded region determined from (*79*) for $P_{H_{2}O}$ < 0.32 bars].

This petrogenetic history may have promoted magmatic devolatilization upon crystallization, which may have in turn driven serpentinization. As an incompatible component, H_2_O in a crystallizing melt will increase in concentration until, depending on pressure, temperature, and melt composition, it reaches saturation and liberates a free aqueous phase (*50*). The H_2_O concentration of Máaz formation melts is not well constrained (*12*), but Schmidt *et al.* (*12*) showed that the relatively low magmatic SiO_2_ concentrations and high temperatures could have generated an assemblage lacking hydrous phases such as biotite and amphibole even if the initial H_2_O concentration was as high as 1 wt %. Nevertheless, a simple examination of magmatic H_2_O solubility suggests that during or shortly after crystallization, the release of a free aqueous fluid phase may have been likely. Figure 8 shows H_2_O concentration as a function of crystallization at different initial H_2_O contents derived from different degrees of partial melting and initial H_2_O concentrations (Fig. 8). In general, these relationships show that H_2_O saturation in the melt would have been reached across a wide range of initial H_2_O contents, especially if the pressure during crystallization was low. Although the emplacement pressure is not precisely constrained, substantial variability in crystal size between individual units of the Máaz formation, possible vesicles, chains of pits, lobate structures, and morphology and distribution across the Jezero crater floor suggests an extrusive origin and emplacement as a series of lava flows (*12*, *15*, *22*, *28*, *29*). Figure 8, however, does not account for intracrustal magmatic processes that may have driven further H_2_O enrichment before melt extraction. For example, extensive fractional crystallization during magma storage under higher pressure conditions within the crust [perhaps as high as 1 to 2 kbar; (*12*)], where H_2_O is highly soluble, may have caused magmatic H_2_O concentrations to increase substantially. Subsequent eruption and depressurization of this melt would have caused cooling, crystallization, and H_2_O solubility to decrease, ultimately resulting in the formation of a free aqueous phase. This aqueous phase would be expected to rise buoyantly through, and interact with, overlying crystals and melt, thus driving serpentinization of fayalitic olivine. Given extensive evidence that magmatic volatiles derived from the martian interior also contained elevated chlorine (*51*, *52*), this may provide a mechanism for Cl enrichment associated with greenalite/hisingerite-rich regions of the Máaz formation abraded targets (figs. S28 and S27 and table S1).

Consistent with these expectations, terrestrial systems that preserve isotopic evidence for melt devolatilization and serpentinization of ferroan olivine, such as the Duluth Complex of Minnesota, USA, bear close mineralogical and textural resemblance to Máaz formation abraded targets (*9*, *38*, *53*–*55*). Similar to the Máaz formation, ferroan gabbros intruded within the crust commonly exhibit selective serpentinization of olivine (*9*, *38*, *55*) and relatively minor alteration of plagioclase and pyroxene. In these systems, the extent of serpentinization is highly variable at small spatial scales and is also accompanied by intimate textural and geochemical association between olivine and secondary products (*9*, *38*, *55*), including the transfer and accumulation of Mn to secondary Fe-rich 1:1 layer silicates and/or magnetite (*9*, *38*, *54*, *55*), as observed in Máaz formation abraded targets (Figs. 2 to 5).

### Implications for early martian climate and habitability at Jezero crater

In situ evidence for serpentinization within the Máaz formation informs several aspects of local- and planetary-scale habitability on early Mars. Because electrons liberated from the oxidation of Fe(II) in olivine are transferred to H_2_O, serpentinization is typically associated with substantial H_2_ generation (*5*, *9*, *40*). Compared to Mg-rich counterparts, the serpentinization of ferroan olivine produces approximately five times as much H_2_ for a given degree of serpentinization, as measured by the total H_2_O content (*9*). Thus, the observation of structural Fe(III) hosted in both magnetite and in Fe-rich 1:1 layer silicate (with the latter reflected by the 2.28-μm absorption in SuperCam-IR data; Fig. 6) associated with serpentinized olivine provides evidence for H_2_ production at the early martian surface.

In contrast to other mechanisms that may have promoted serpentinization at the early martian surface, in situ data from the Máaz formation allow for the possibility that the timing and spatial extent of serpentinization (and associated H_2_ production) may have been closely linked to hydrous magmatism and therefore at least partly decoupled from the availability of surface or near-surface liquid water. Although the degree of devolatilization-driven serpentinization would have been dependent on the total volatile load of the parent magma, cooling rate, eruptive style, and C/H ratio, petrologic constraints and remotely sensed geochemical data suggest that Noachian magmas may have been more H_2_O rich than younger counterparts, possibly owing to a lack of an effective mechanism to rehydrate the martian mantle (*56*, *57*). This in turn raises the possibility that serpentinization associated with hydrous magmatism may have been most frequent earlier in martian history, consistent with orbital observations of widespread detections of Fe(III)-phyllosilicates in highly magnetized regions of the most ancient martian crust (*9*). Given that percent-level atmospheric H_2_ concentrations lead to an enhanced greenhouse effect in CO_2_-dominated atmospheres (*3*), the timing and nature of large-scale hydrous magmatism may have strongly influenced the timing and tempo of surface warming on early Mars.

Serpentinization also may have helped shape the habitability of aqueous environments on early Mars more generally and within Jezero crater specifically. On Earth, serpentinizing systems maintain redox disequilibria among C, N, Mn, Fe, and S species, in turn supporting diverse microbial metabolisms (*58*, *59*). In addition, H_2_ generated from serpentinization can reduce CO_2_ to methane and/or CO to facilitate the abiotic synthesis of organic compounds with diverse molecular functionality (*59*, *60*); these synthetic pathways operated within the ancient martian crust (*4*) and potentially within Jezero crater. Because geochronological constraints delineate a broad temporal interval within which fluvial and igneous activity across the Jezero crater floor both occurred (*61*, *62*), serpentinization may have repeatedly supplied both H_2_ and/or organic compounds to diverse aqueous environments within Jezero crater. As potential archives of such astrobiologically significant processes, samples extracted from the Jezero crater floor and cached by the Perseverance rover are therefore among the highest priority targets for potential Mars sample return.

## MATERIALS AND METHODS

### Quantification of PIXL XRF data

Analysis of PIXL XRF data was performed using the XRF visualization and analysis tool PIXLISE (*63*, *64*). Elemental quantification of PIXL data used an iterative fundamental parameters physics-based model (*65*), which converts x-ray peak intensity into elemental concentration. However, PIXL elemental quantifications are calibrated for flat surfaces which are perpendicular to the x-ray source. Natural surfaces oriented at an angle toward one of PIXL’s detectors and away from the other result in summed spectra that are less intense than those produced from flat surfaces. As a consequence, this underestimation will scale with the degree to which natural surfaces are angular or “rough.” An additional factor that can influence XRF quantification relates to overlap between diffraction and fluorescence peaks; this would result in overestimation of associated oxide or element abundances.

The effects of both roughness and diffraction were estimated and corrected for by producing an approximate estimate of the orientation of the local rock surface relative to the detector by examining the difference between spectra collected by the two detectors (*12*, *66*). The resulting predicted difference in fluorescence peak intensities for each detector was compared with the actual difference observed. If the predicted and observed intensities were similar, then the associated peak was assumed free of diffraction overlap, and thus abundances were used to correct for estimated orientation. If the intensities differed from that predicted by surface orientation, then abundances were estimated from the detector not affected by diffraction overlap and corrected for the estimated orientation. The specific correction routines and their derivation are explained in detail in (*12*)

### Identification and isolation of Fe-Si material in PIXL scans

As shown in Figs. 2 to 5, the Fe-Si material exhibits a distinctive composition compared to the remaining XRF spot analyses in each PIXL XRF scan. To facilitate further analysis and interpretation of the Fe-Si material across these four scans, we refined the Fe-Si population simply by excluding XRF spot analyses which were influenced by mixing and contamination with Fe-Ti oxide minerals [interpreted as igneous in origin (*12*)] and S-bearing minerals.

Refinement of XRF spot analyses comprising the Fe-Si material first involved selection of XRF spot analyses that deviated toward the (Fe + Mg) apex of the (Ca + Na + K), Si, and (Fe + Mg) ternary and were thus controlled by minimal mixing with primary silicate phases such as feldspars and pyroxene. Then, within this region, XRF spot analyses were rejected that exhibited SO_3_ concentrations greater than 8.0 wt %. This cutoff was chosen on the basis of binary mixing relationships between FeO and SO_3_ oxides present in each of the four scans, in particular the Bellegarde scan (fig. S1). FeO-SO_3_ plots show that Guillaumes, Montpezat, and Alfalfa are generally associated with relatively low SO_3_ concentrations (less than 1 wt % in Guillaumes and Montpezat and less than 4 wt % in Alfalfa). Bellegarde, however, exhibits higher SO_3_ concentrations due to locally high accumulations of S-bearing minerals which appear to be hosted within void space (*15*). One void in particular also contains a Fe-Si material toward the outer edge and a high S-bearing material toward the center, which is manifested in PIXL XRF data as a clear linear trend between the high FeO/low SO_3_ material and the high SO_3_/low FeO material. Thus, the SO_3_ cutoff was chosen on the basis of extrapolation of the high SO_3_/low FeO material to average SO_3_ concentrations present in the remainder of the scan. Figure 3 shows, however, that minor mixing between S-bearing minerals and the Fe-Si material is present within this population, as some points exhibit Ca + Na + K ternary values higher than other scans (due to mixing with small abundances of Ca-S–bearing material). These points are located in close spatial association with locally high accumulations of S-bearing minerals in the Bellegarde scan (fig. S3).

Next, XRF spot analyses were rejected on the basis of contamination by Fe-Ti oxides. We adopted a cutoff whereby analyses with TiO_2_ concentrations greater than 3.0 wt % were rejected, which was determined by FeO and TiO_2_ binary relationships for the Bellegarde target (fig. S2). FeO-TiO_2_ plots show that all targets contain Fe-Ti oxide minerals, contamination from which can be readily identified by linear trends between high FeO/high TiO_2_ and low FeO/low TiO_2_ materials. This effect can be removed by extrapolating to very low TiO_2_ values (fig. S2). Although Fe-Ti oxides are present in all targets, Guillaumes, Montpezat, and Alfalfa show that the Fe-Si material is distinct from these regions in their high FeO and low TiO_2_ compositions; this is also manifested by distinct spatial distributions of the Fe-Si material compared to Fe-Ti oxides (fig. S4). However, the Bellegarde target exhibits more complicated behavior. Here, XRF spot analyses show a clear linear trend controlled by Fe-Ti oxide mixing, but the data also exhibit mixing between Fe-Si material and Fe-Ti oxides. To exclude the effects of this mixing, we extrapolated to 3.0 wt % TiO_2_, reflective of average TiO_2_ concentrations in the remainder of the scan.

The resulting populations of XRF spot analyses corresponding to the Fe-Si material referred to here in the main text were then used to facilitate comparison based on MCC reflectance properties (Fig. 7 and fig. S10). The resulting average compositions of these populations are given in table S1.

### Back-reflected x-ray diffraction

The PIXL instrument collects diffraction peaks in its two energy-dispersive detectors, which enables energy-dispersive x-ray diffraction data to be collected and analyzed (*24*). There are two cases in which a crystalline material may not produce diffracted x-rays detected by PIXL. The first case is where a material is either amorphous or has very small crystal size. Quantitative analysis of the effect of crystallite size on diffraction peak intensity was performed through the analysis of halite (NaCl) crystals of varying sizes with a PIXL Breadboard instrument at the Jet Propulsion Laboratory (JPL); this suggests a minimum detectable crystal size on the order of less than 45 μm (*24*). The second case is where no crystal lattice is oriented such that x-rays are diffracted back to PIXL’s detectors. Experimental data and numerical modeling have shown that PIXL has an 80% probability of detecting diffraction data in either of its two detectors, if a crystalline coherent scattering domain of sufficient size is present (*24*). This suggests that where no diffraction peaks are detected by PIXL, the lack of diffraction is likely due to material characterized by crystalline domains which are too small to produce sufficient diffracted x-rays for detection.

### PIXL MCC constraints on chromophore minerals

In addition to its microfocus XRF, the PIXL instrument includes the MCC, two structured light illuminators, and flood light illuminators (FLIs) (*67*). Multispectral data are acquired by operating these systems in concert. The FLI provides an active light source composed of 24 light-emitting diodes evenly distributed into four color channels (where wavelengths correspond to peak maxima and uncertainties correspond to half width at half maximum): UV (385 ± 4 nm), blue (447 ± 9 nm), green (523 ± 13 nm), and NIR (723 ± 10 nm) (*67*). The optics of the FLI are designed and calibrated such that the uniformity of illumination and emitted power are optimal at a standoff distance of 25.5 mm. The PIXL optical fiducial subsystem (OFS) collects multispectral data by capturing a grayscale MCC image, while the FLI is emitting light of a single color channel at a given time. This collection of four images is referred to as a color stack. To compare the individual images in the color stack and enable color imaging and multispectral analysis, a radiometric correction procedure outlined in (*67*) was utilized. In general, this procedure yields data products in excellent agreement with bidirectional reflectance data acquired in the laboratory (*67*). As a consequence, reflectance band ratios of mineral standards determined through preflight calibration and breadboard instruments correspond closely to those determined from laboratory bidirectional reflectance data.

The major Fe(III) oxide minerals exhibit distinct reflectance properties from most Fe-silicate minerals. Although individual laboratory measurements of absolute reflectance can differ as a function of sample crystallite size and measurement conditions (including sample topography), comparisons can be made across similar sample properties and measurement conditions, with samples of well-characterized crystal structure and chemistry. Figure S15 shows laboratory reflectance data acquired on four Fe oxide minerals (from the reflectance experiment laboratory (RELAB) spectral database], all of which are finely crystalline (consistent with PIXL diffraction constraints on a crystallite size less than 45 μm). In general, these data show that Fe(III) oxide minerals are characterized by high reflectance in the NIR compared to shorter wavelengths and thus have high NIR/UV ratios. The exception to this is magnetite, which, even in finely crystalline form, exhibits low NIR/UV ratios due to low absolute reflectance values across the UV/vis/NIR (fig. S15).

To examine and verify the sensitivity of PIXL’s MCC instrument and thus measured NIR/UV ratios to Fe(III) oxide chromophore minerals, we examined data acquired from the Pignut Mountain target, for which PIXL MCC and XRF data were acquired on sol 463. Before the acquisition of PIXL data, this target was analyzed by the SuperCam laser induced breakdown spectroscopy (LIBS) instrument, which removed natural dust accumulated on the rock surface (fig. S14), thus offering an ideal test case to examine the spectral properties of surfaces which contain Fe(III) oxide minerals. Although the measured compositions of martian surface dust exhibits minor differences from locality to locality, compositions are generally remarkably consistent between localities thousands of kilometers apart (*68*). Magnetic measurements, coupled with M ossbauer spectroscopy, on dust accumulated on the Mars Exploration Rover magnets have shown that the dominant mineralogy is composed of ferrous Fe(II)-bearing silicates and Fe-Ti oxides and smaller proportions of a poorly crystalline Fe(III) oxide component most consistent with mixtures containing ferrihydrite (*68*). XRF data acquired on dust-rich and dust-poor areas of the Pignut Mountain scan (table S2) indicate minor differences in bulk composition with the exception of lighter *Z* elements including Mg and S; Fe shows negligible change. These compositional differences likely relate to differing penetration depths of x-rays as a function of their energy (*31*), with lighter *Z* elements exhibiting shallower penetration depths and thus more sensitive to the effects of surface dust. Nevertheless, the data acquired on Pignut Mountain thus indicate that minor concentrations of chromophore Fe(III) oxide minerals (as inferred from compositional and mineralogical analyses of martian dust to date and consistent with compositional data in table S2) carry a marked effect on reflectance properties, specifically the NIR/UV ratio, as measured by PIXL’s MCC.

In contrast to Fe(III) oxide minerals shown in fig. S15, fig. S16 shows laboratory reflectance data acquired on three Fe-silicate minerals (from the RELAB spectral database), two of which represent members of the greenalite-hisingerite solid solution and one sample of fayalitic olivine. Although the spectral properties show variation as a function of the Fe(II)/Fe(Total) ratio, these silicates are characterized by much lower NIR/UV ratios than Fe(III) oxides.

Together, MCC reflectance data on three Máaz formation abraded targets (Bellegarde, Montpezat, and Alfalfa) show that the Fe-Si material generally exhibits low NIR/UV ratios (Fig. 7), consistent with substantial proportions of materials such as olivine, Fe-rich 1:1 layer silicates, and/or magnetite. Although these results are inconsistent with substantial proportions of Fe(III) oxide minerals, small concentrations of these phases cannot be ruled out. Although Fig. 7 and figs. S11 to S13 show that the highest NIR/UV ratios measured by PIXL’s MCC do not correspond to the Fe-Si material, some Fe-Si material XRF spot analyses do exhibit relatively high NIR/UV ratios, which may result from small concentrations of Fe(III)-bearing phases, in particular for the Alfalfa and Bellegarde targets; these results are consistent with Mastcam-Z analyses of these abraded targets (*29*). However, PIXL XRF data show that XRF spot analyses of Fe-Si material that exhibit relatively high NIR/UV ratios are almost always associated with elevated Cl content. This strong association between Fe and Cl, across all targets where Fe-Si material has been identified, suggests a genetic link between the two elements, likely in the form of now oxidized discrete ferrous hydroxychloride phases (*46*) or double-layered hydroxides such as pyroaurite or iowaite (*47*), which commonly form during serpentinization of ferroan protoliths.

### Clustering and Gaussian mixture modeling

Clustering and Gaussian mixture modeling was carried out using an adapted workflow developed by Tung *et al.* (*35*) named SIGMA which uses several different classes of machine learning algorithms to automatically sort the data points into separate clusters based on their elemental abundances. The workflow, which is implemented in Python, first involves representing the dataset in a reduced dimensional space before applying clustering to group together points with similar elemental signatures. Different combinations of reduction methods and clustering were tested before a final workflow with set hyperparameters for reduction was chosen. For dimensionality reduction, uniform manifold approximation and projection (*69*) was applied with *n*-neighbors set to 15, “mindist” set to 0, and a Euclidean distance metric. This parameter combination provided better comparability between datasets and good preservation of local structure while retaining continuity between clusters. This dimensionality reduction was carried out on the elemental abundances provided by PIQUANT. For clustering, the scikit-learn implementation of MeanShift clustering (*70*) was used with bandwidths set depending on the number of clusters visually observable in the latent space resulting from dimensionality reduction (Bellegarde set to 2.5, Alfalfa to 2.6, Montpezat to 2.8, and Guillaumes to 2.1); this is generally dependent on the compositional complexity of each sample.

### Compositional differences between nontronite and greenalite/hisingerite

To quantify compositional domains for different groups of Fe-rich low-Al phyllosilicates, we compiled electron microprobe microanalytical data on well-chararacterized mineral specimens from the literature. We only chose data where independent constraints on mineralogical composition and/or crystal structure were available (i.e., through spectroscopic analysis, x-ray diffraction, or high-resolution transmission electron microscopy). This allowed us to identify six distinct compositional domains: chlorite, berthierine, saponite, nontronite, hisingerite, and greenalite (fig. S6). Literature sources are Greenalite-hisingerite (*10*, *37*, *38*, *71*); Nontronite-saponite (*72*–*74*); chlorite (*75*); and berthierine: (*76*).

In comparison to these six compositional domains, the composition of Fe-Si material across all four abraded targets precludes substantial concentrations of berthierine and/or chlorite. In addition, this comparison shows that the Fe-Si material is most consistent with mixtures containing substantial proportions of hisingerite-greenalite as opposed to nontronite (or saponite), the latter of which being characterized by a lower Fe/Si ratio. Few XRF spot analyses corresponding to the Fe-Si marterial lie within the nontronite-saponite region (fig. S8). Mn concentration serves as the more diagnostic discriminator between hisingerite and nontronite. To assess the relative Mn concentrations of hisingerites and nontronites, we compiled literature sources where nearly monomineralic composition has been independently demonstrated, to avoid contamination. The result of this comparison includes nontronite and hisingerite derived from a variety of formation environments spanning a large range in temperature and local redox conditions (fig. S7). In combination with data from the Fe-Si material showing the absence of diffraction and enrichment in Mn (fig. S9), this strongly supports an important role for hisingerite, although the presence of nontronite cannot be ruled out [nontronite commonly occurs as a product of the serpentinisation of Fe-rich protoliths on Earth; (*55*)].

### Laboratory micro-XRF measurements of Duluth Complex serpentinites

To examine the effects of mixing between finely crystalline minerals in PIXL data, we acquire laboratory micro-XRF data from polished slabs extracted from serpentinized rocks from the 1.1 Ga Duluth Complex of the North American Mid-Continent rift. Samples were collected by B. Tutolo (University of Calgary, Canada) from the Bardon Peak outcrop (46°40.745′N, 92°14.180′W) of the troctolitic series within the Duluth Complex (*9*). Corresponding thin sections to the slabs were examined with polarized light microscopy and revealed microfracture-hosted alteration within olivine-rich domains. The microfractures are dominated by Fe-rich 1:1 layer silicate (greenalite-hisingerite solid solution) and magnetite. The magnetite occurs principally as small crystals on the order of 20 to 50 μm in size, but these crystals accumulate locally to generate larger magnetite-rich domains on the order of several millimeters in size (fig. S17).

Micro-XRF analyses were undertaken in the Department of Geology and Geophysics at Texas A&M University using a HORIBA XGT-7000 x-ray fluorescence analytical microscope. Micro-XRF data were acquired across full polished billets 30 mm by 24 mm in size at multiple x-ray beam diameters. To facilitate comparison with PIXL, the resolution of individual spot analyses was downgraded to ~120 μm.

The results of the analyses are shown in fig. S18. These data not only illustrate clear mixing between the principal rock-forming silicate phases olivine (with minor plagioclase) but also generate a data cloud that closely corresponds to compositions characterized by mixing between Fe-serpentins (greenalite-hisingerite soid solution) and olivine (fig. S18), as observed in PIXL XRF data acquired from four Máaz formation abraded patches. The data also show that few points extend beyond olivine end-member compositions in (Ca + Na + K), Si, and (Fe + Mg) ternary space, as is the case for Guillaumes, Bellegarde, and Montpezat. This effect arises because of mineral mixing at a scale that is similar to, or smaller than, the x-ray beam size. When the x-ray beam size is smaller, a larger number of spot analyses capture magnetite-rich regions.
