# Supplementary material for: In situ evidence for serpentinization within the Máaz formation, Jezero crater, Mars
Source: Sci Adv. 2025 Jul 2;11(27):eadr8793. doi: 10.1126/sciadv.adr8793 (PMC12219501; doi:10.1126/sciadv.adr8793)
Supplement: Supplementary file 1 — Figs. S1 to S28 Tables S1 and S2 [file sciadv.adr8793_sm.pdf]

Science Advances
AAAS

Supplementary Materials for

In situ evidence for serpentinization within the Máaz formation, Jezero crater, Mars

Nicholas J. Tosca *et al.*

Corresponding author: Nicholas J. Tosca, njt41@cam.ac.uk

Sci. Adv. **11**, eadr8793 (2025)
DOI: 10.1126/sciadv.adr8793

This PDF file includes:

Figs. S1 to S28
Tables S1 and S2

Supplementary Figures

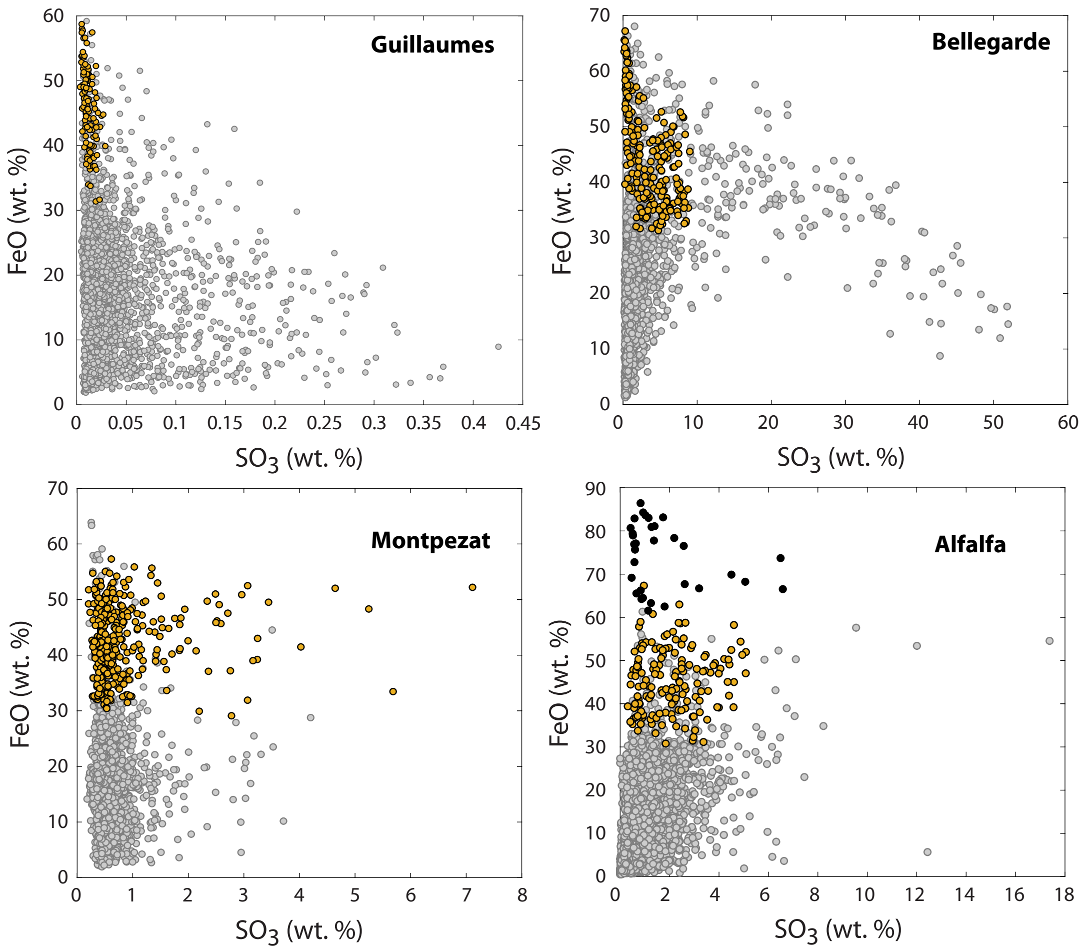

Figure S1: FeO versus SO_3 concentrations for the four Máaz formation abraded targets. Orange circles: individual XRF spot analyses corresponding to Fe-Si material; grey circles: individual XRF spot analyses corresponding to remaining portion of the PIXL scan; black circles: individual XRF spot analyses corresponding to high-Fe material within the Alfalfa target. All XRF data were corrected for surface roughness and diffraction effects as discussed in *Materials and Methods*.

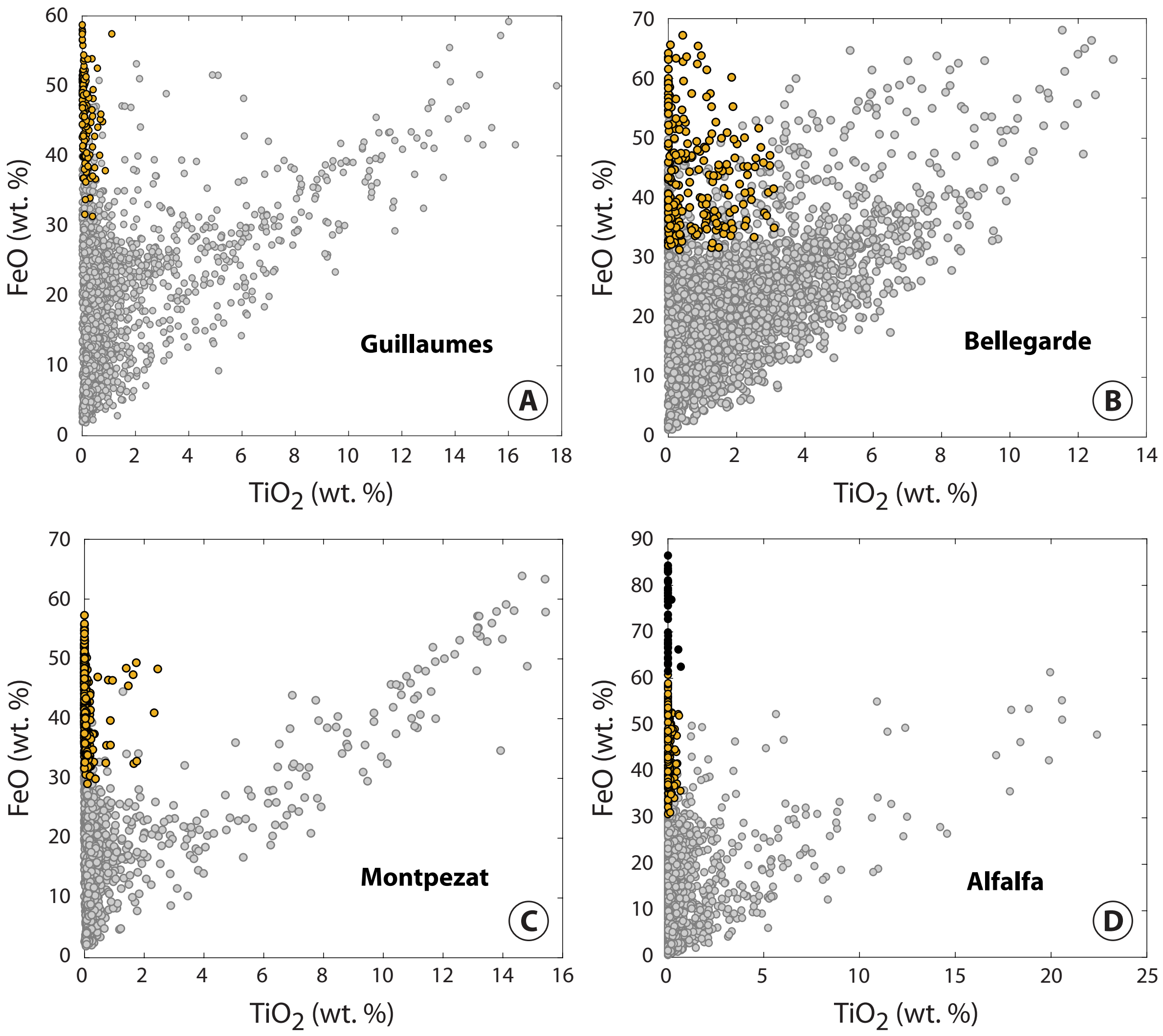

Figure S2: FeO versus TiO_2 concentrations for the four Máaz formation abraded targets. Orange circles: individual XRF spot analyses corresponding to Fe-Si material; grey circles: individual XRF spot analyses corresponding to remaining portion of the PIXL scan; black circles: individual XRF spot analyses corresponding to high-Fe material within the Alfalfa target. All XRF data were corrected for surface roughness and diffraction effects as discussed in *Materials and Methods*.

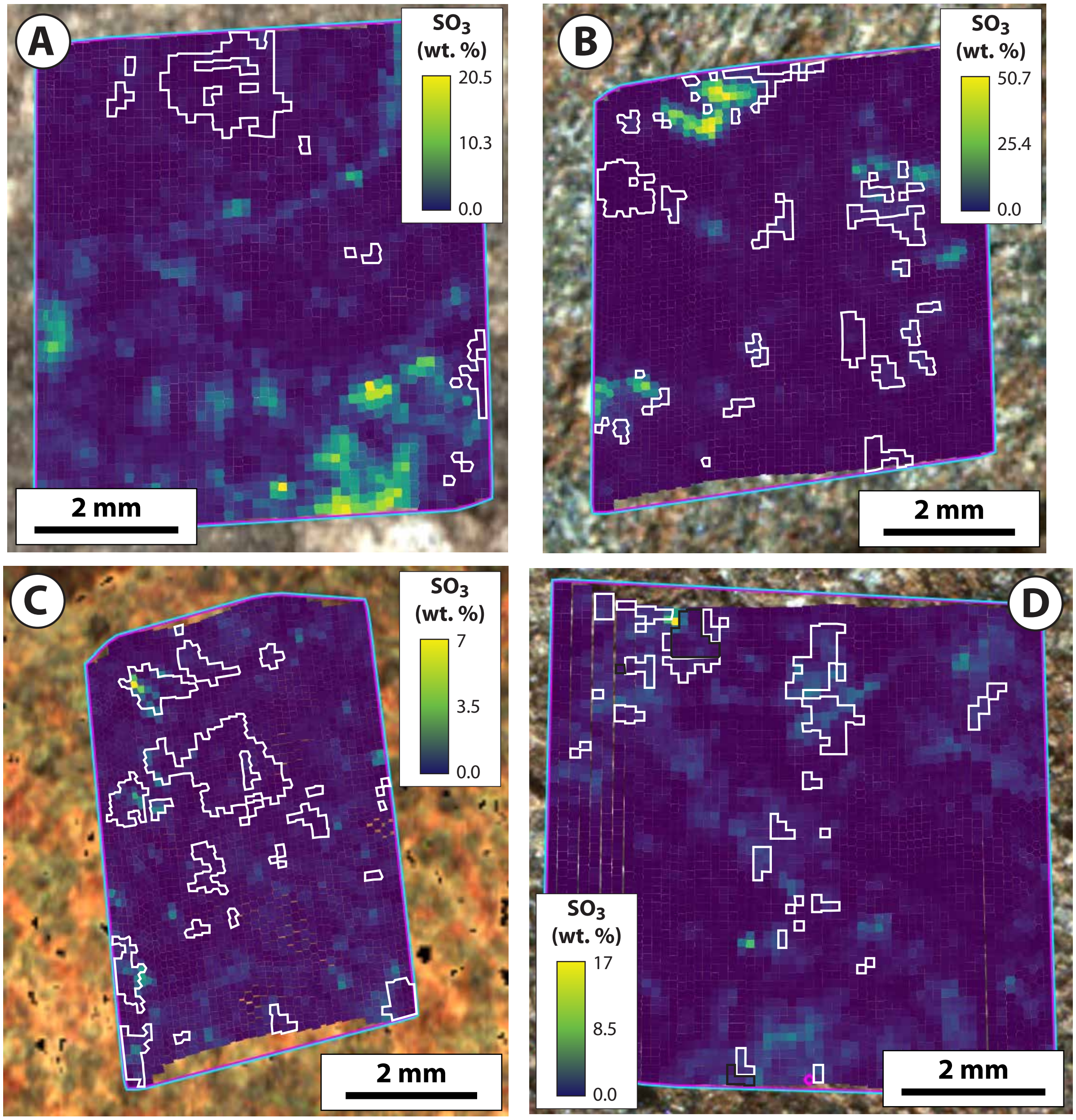

Figure S3: SO_3 abundance maps for the four Máaz formation abraded targets: Guillaumes (A), Bellegarde (B), Montpezat (C), and Alfalfa (D). White regions delineate Fe-Si material, which corresponds to orange points in Figure S1. All XRF data were corrected for surface roughness and diffraction effects as discussed in *Materials and Methods*.

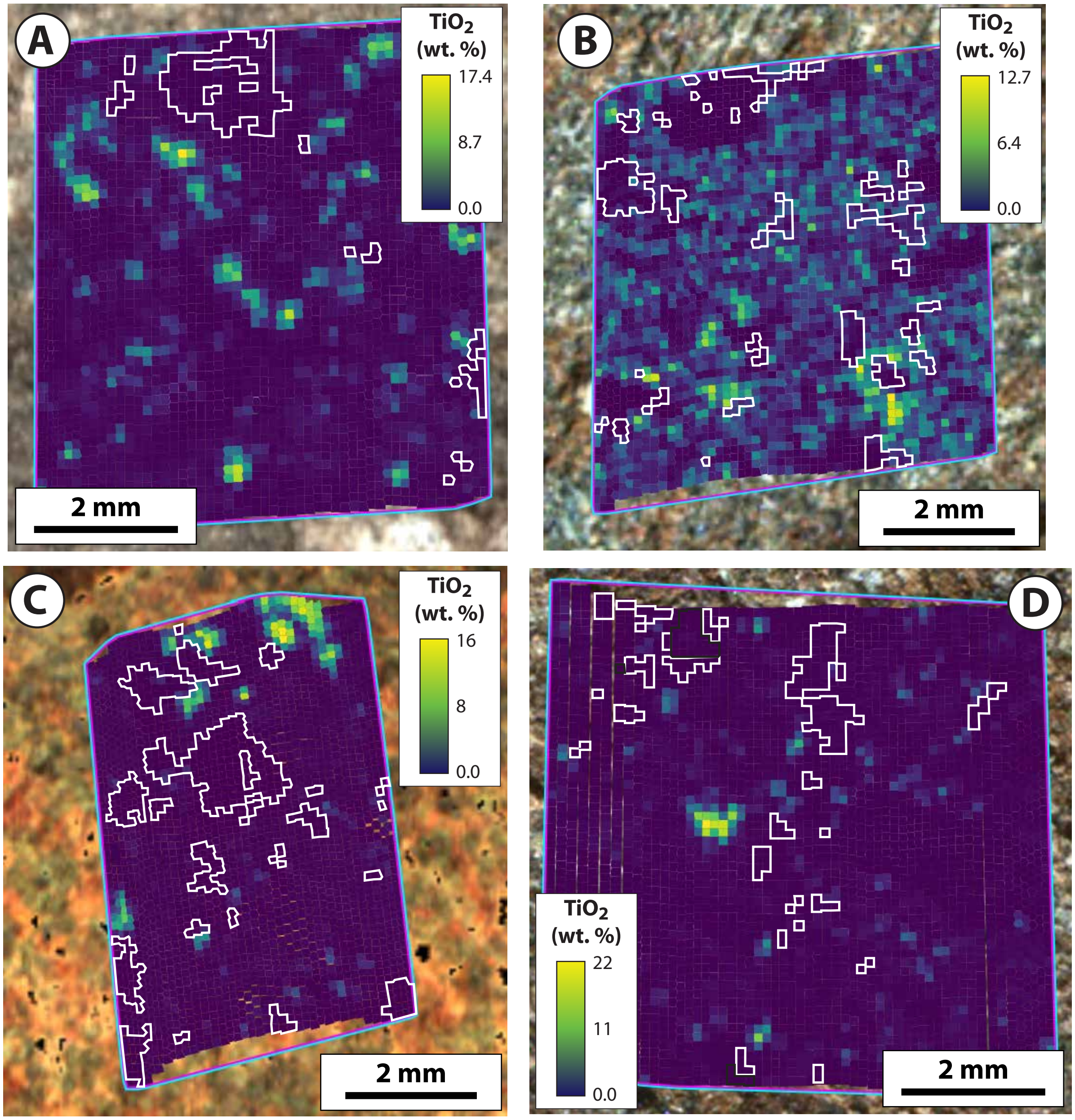

Figure S4: TiO_2 abundance maps for the four Máaz formation abraded targets: Guillaumes (A), Bellegarde (B), Montpezat (C), and Alfalfa (D). White regions delineate Fe-Si material, which corresponds to orange points in Figure S2. All XRF data were corrected for surface roughness and diffraction effects as discussed in *Materials and Methods*.

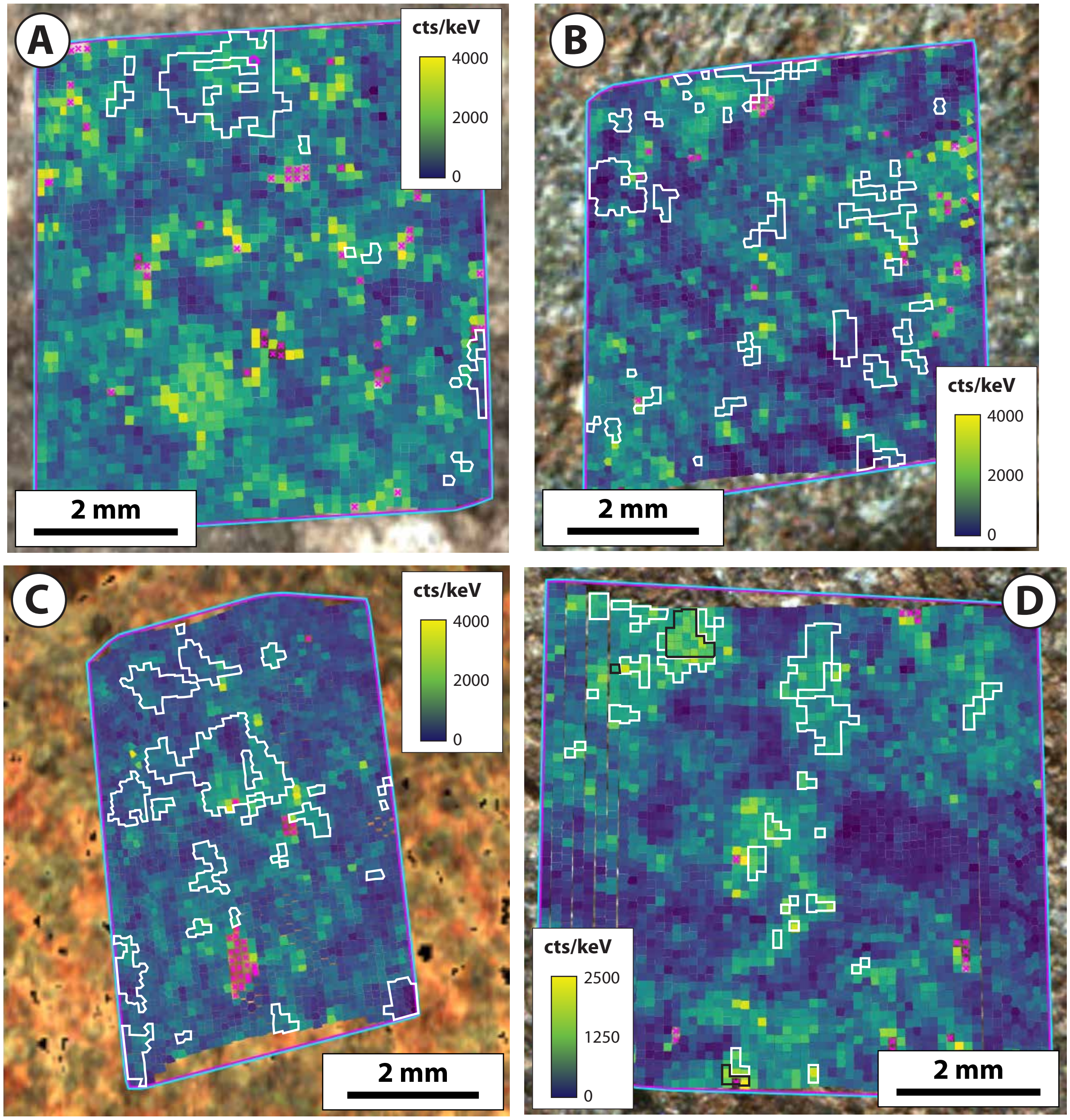

Figure S5: Maps showing the intensity of backscattered X-rays from the Rh L line for the four Máaz formation abraded targets: Guillaumes (A), Bellegarde (B), Montpezat (C), and Alfalfa (D). X-ray intensity is corrected for surface roughness and diffraction effects as discussed in *Materials and Methods*.

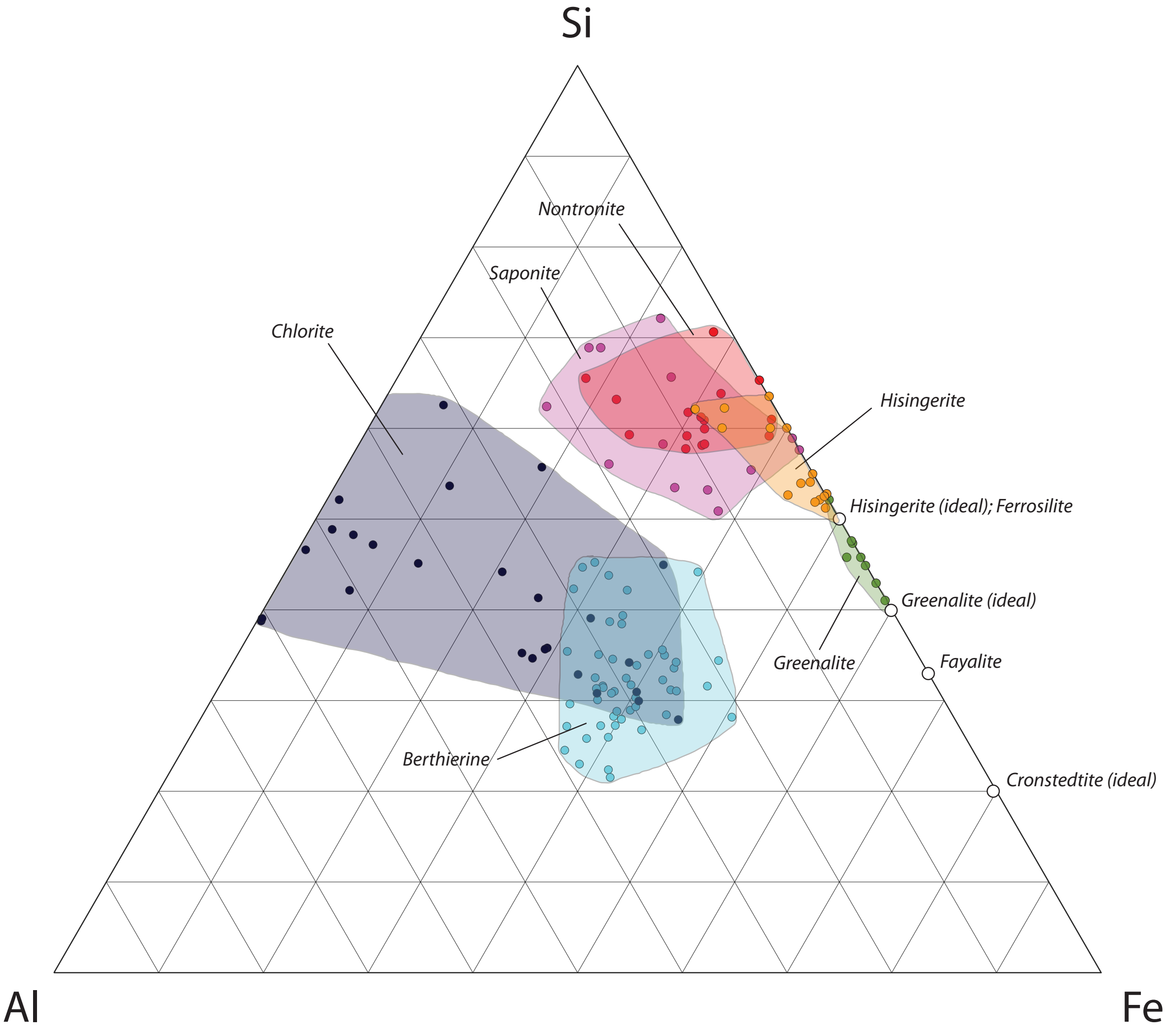

Figure S6: Results of a literature compilation of compositional analyses for Fe-rich, Al-poor phyllosilicates. Literature sources are: Greenalite-hisingerite: (*37, 71, 10, 38*); Nontronite-saponite: (*72, 73, 74*); Chlorite: (*75*); Berthierine: (*76*).

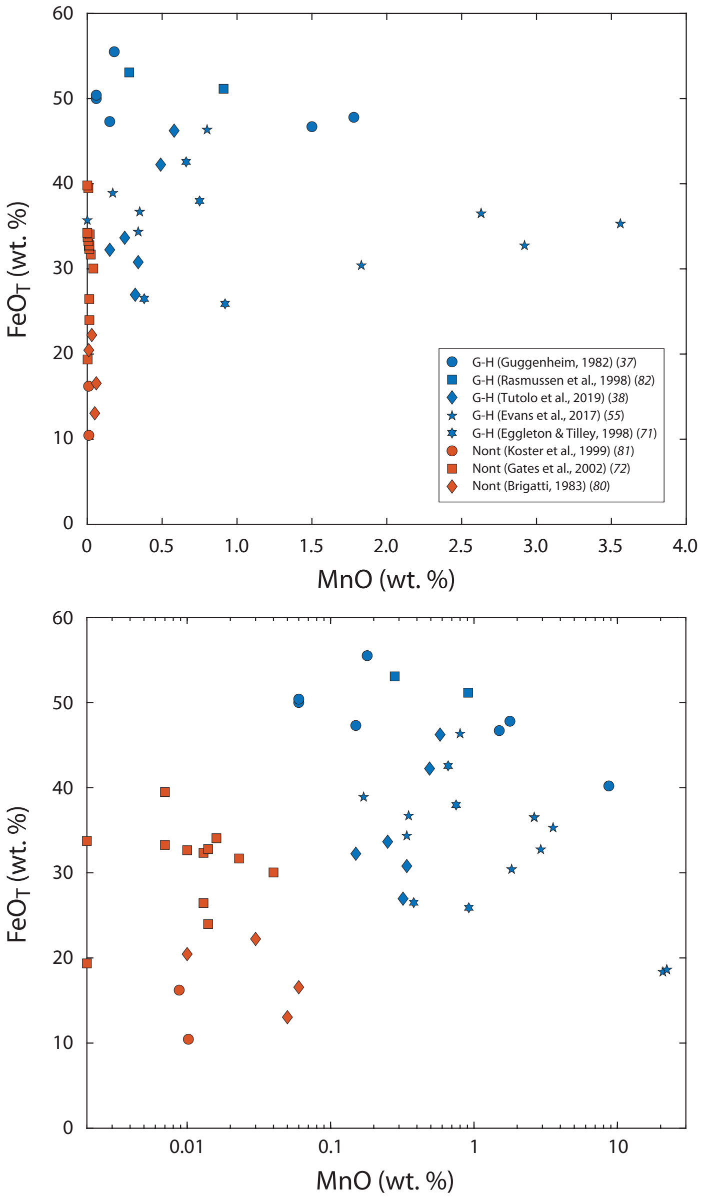

Figure S7: MnO concentrations in monomineralic specimens of greenalite/hisingerite and nontronite sampled from a wide range of formation environments as a function of temperature and oxidation state.

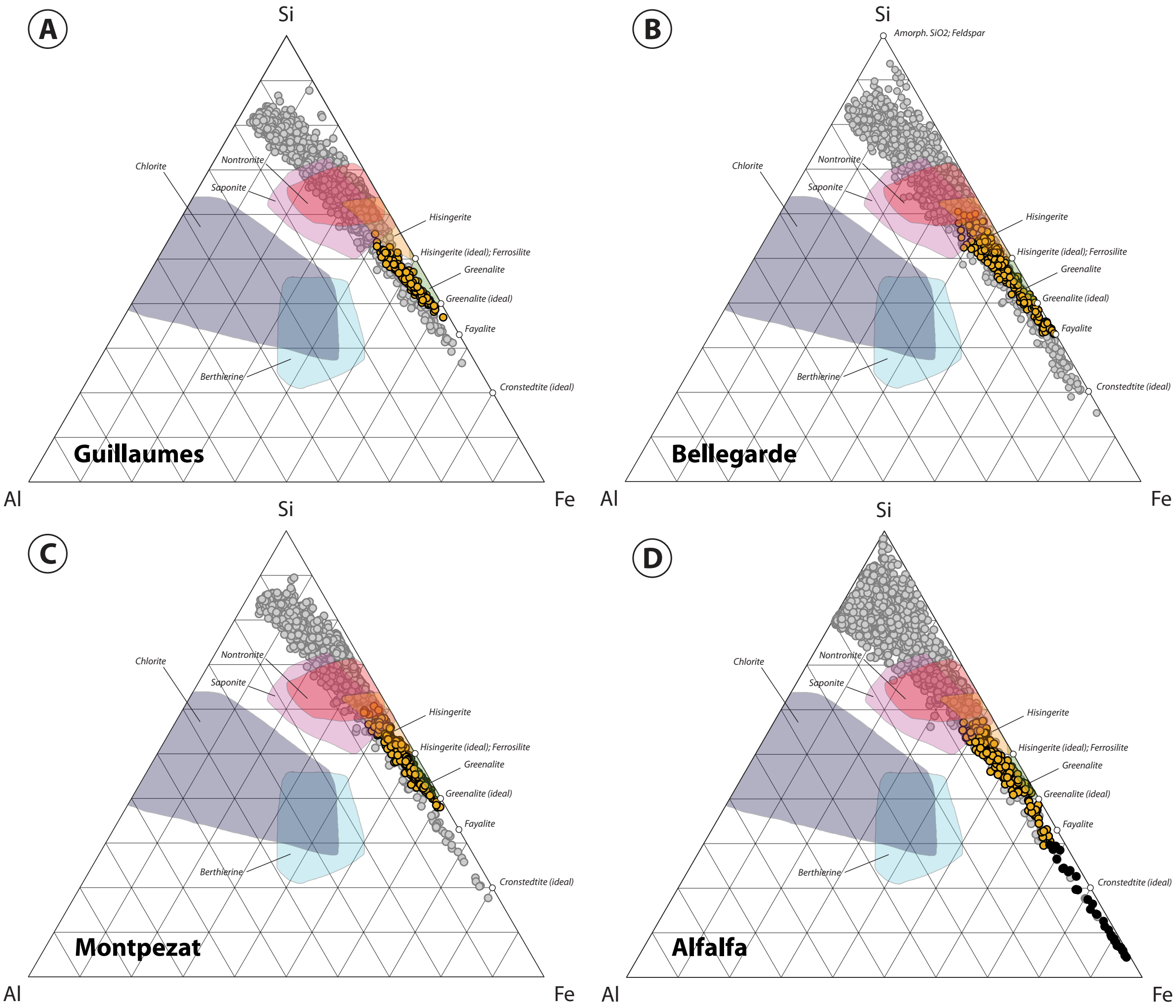

Figure S8: Comparison between compositional domains for Fe-rich, Al-poor phyllosilicates (shown in Figure S6) and PIXL XRF spot analyses for the four Máaz formation abraded targets. Orange circles: individual XRF spot analyses corresponding to Fe-Si material; grey circles: individual XRF spot analyses corresponding to remaining portion of the PIXL scan; black circles: individual XRF spot analyses corresponding to high-Fe material within the Alfalfa target. All XRF data were corrected for surface roughness and diffraction effects as discussed in *Materials and Methods*.

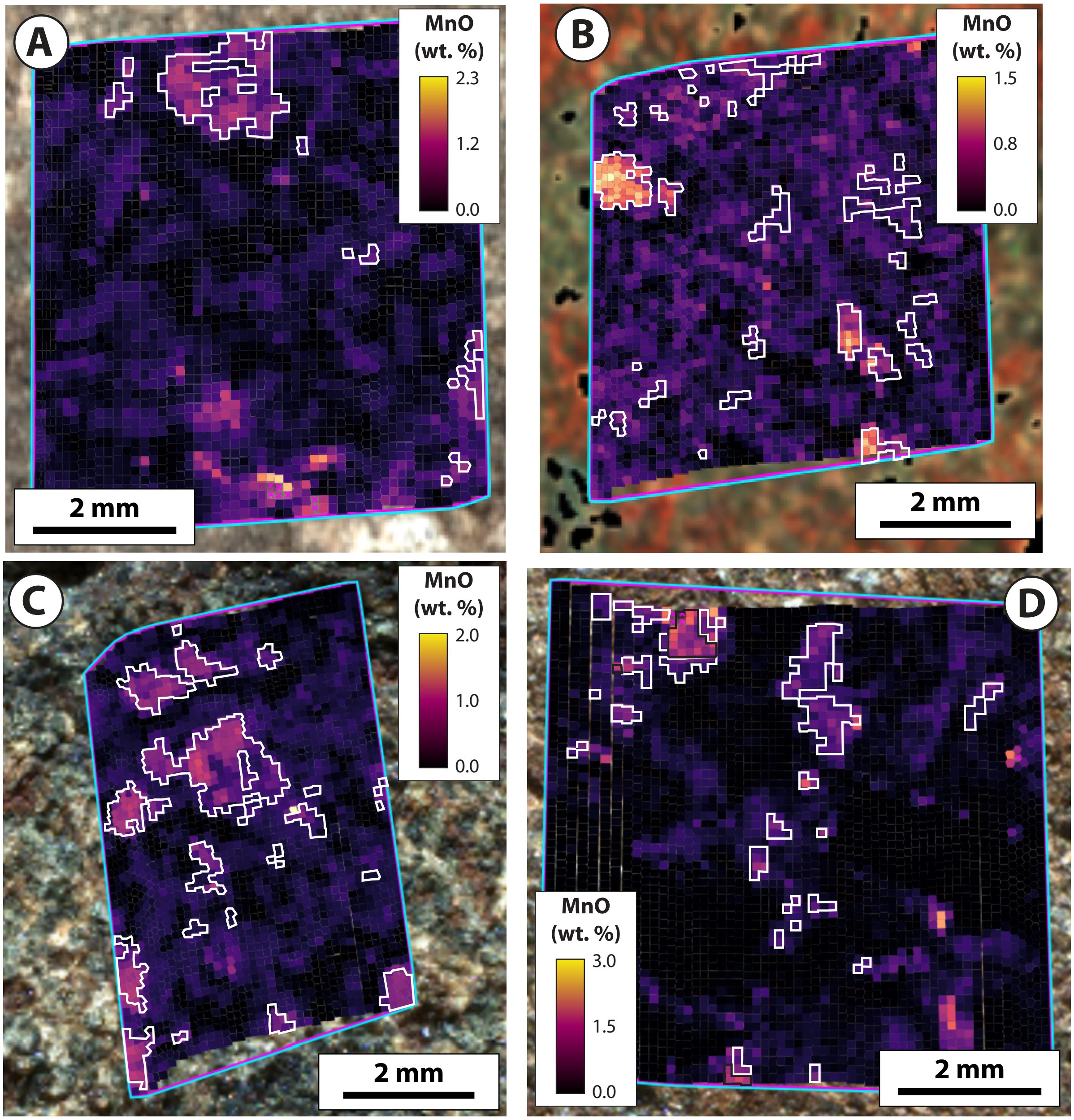

Figure S9: MnO abundance maps for the four Máaz formation abraded targets: Guillaumes (A), Bellegarde (B), Montpezat (C), and Alfalfa (D). White regions delineate Fe-Si material.. All XRF data were corrected for surface roughness and diffraction effects as discussed in *Materials and Methods*.

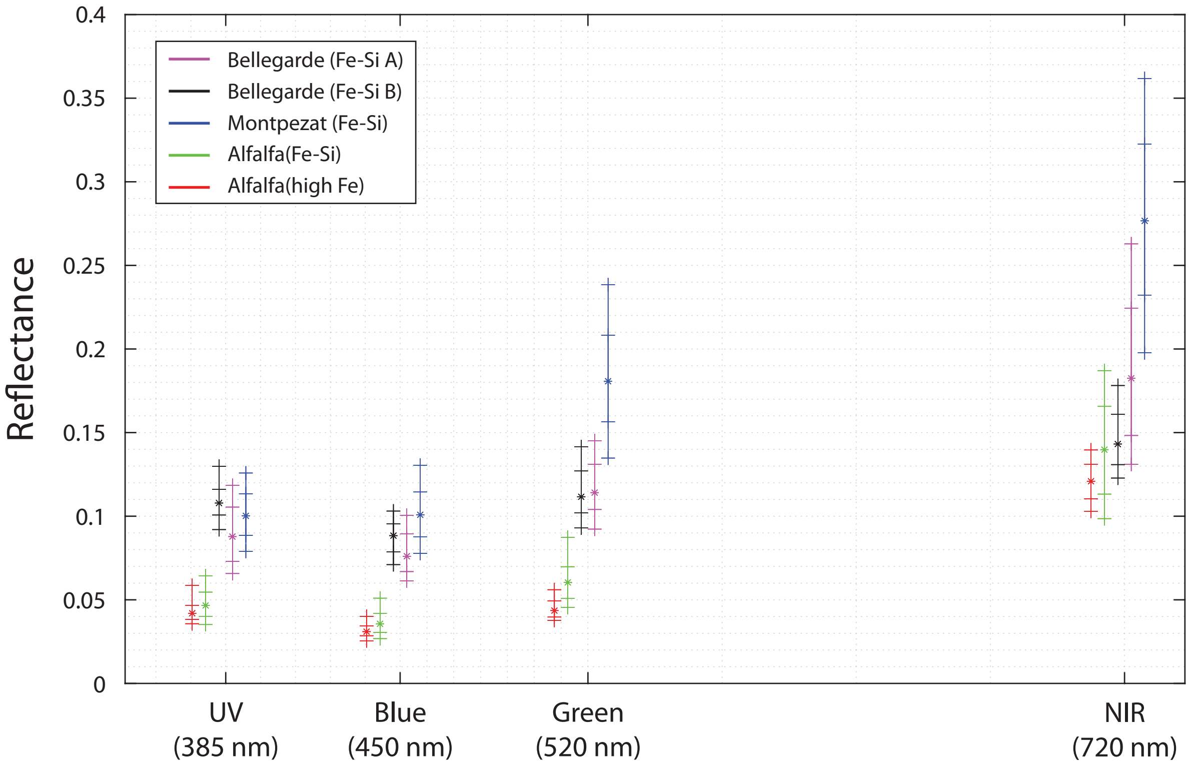

Figure S10: Absolute reflectance data, determined by PIXL's MCC, for three Máaz formation abraded targets.

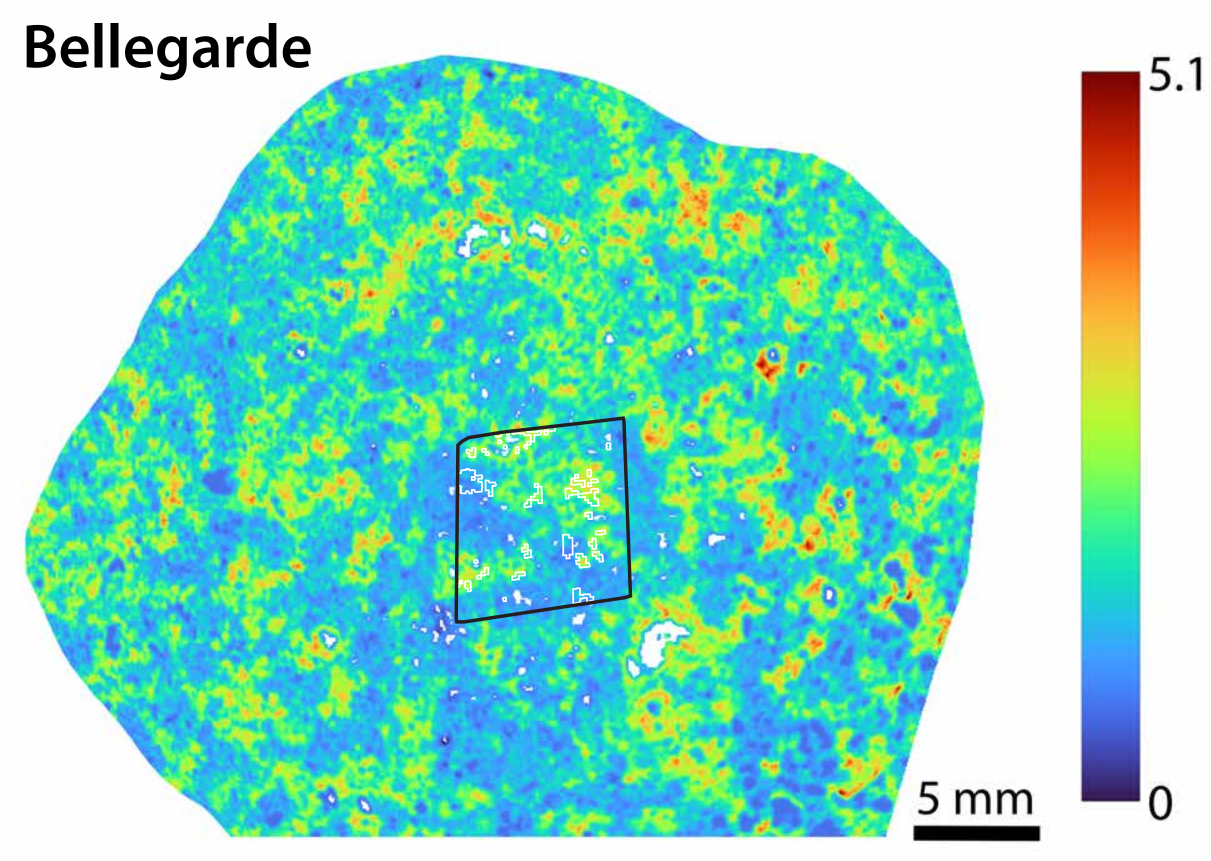

Figure S11: Map of NIR/UV ratio, as measured by PIXL's MCC, for the Bellegarde abraded patch. PIXL XRF scan area shown in black. White regions delineate Fe-Si material, which corresponds to white points in Figure 7. Images were cropped to remove abrasion fines and vignetting.

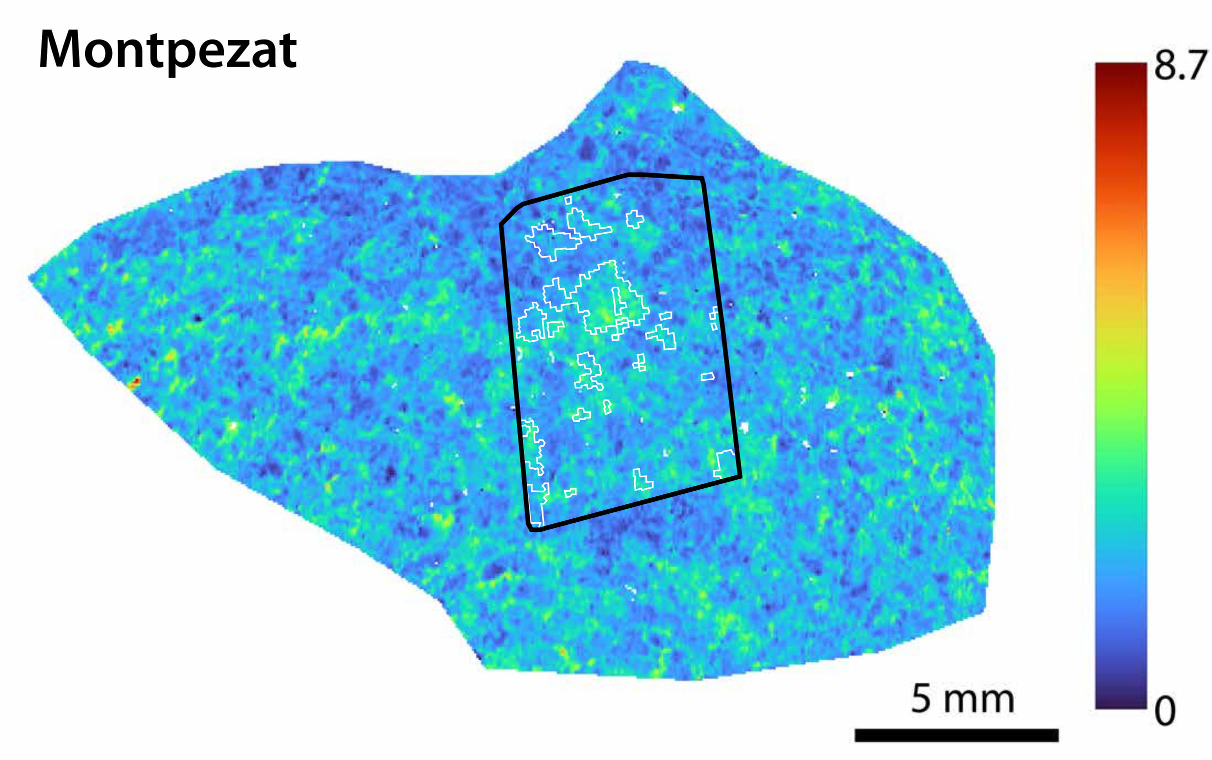

Figure S12: Map of NIR/UV ratio, as measured by PIXL's MCC, for the Montpezat abraded patch. PIXL XRF scan area shown in black. White regions delineate Fe-Si material, which corresponds to white points in Figure 7. Images were cropped to remove abrasion fines and vignetting.

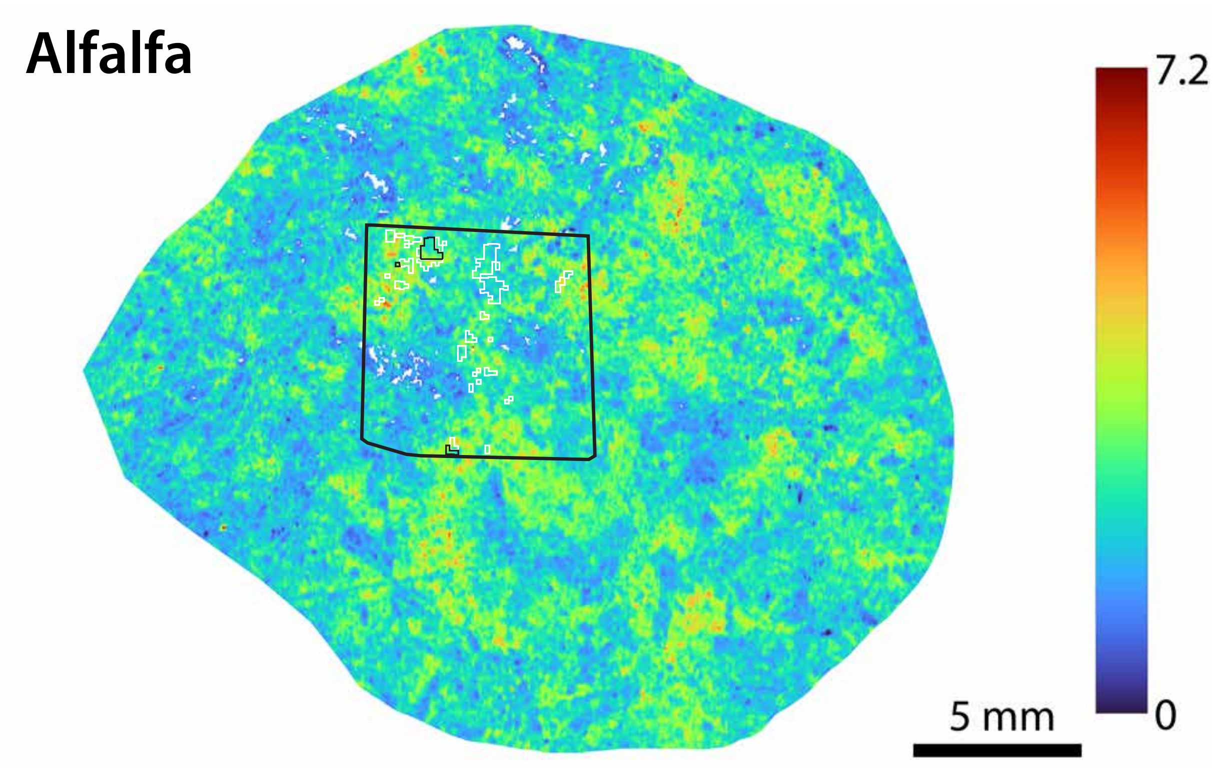

Figure S13: Map of NIR/UV ratio, as measured by PIXL's MCC, for the Alfalfa abraded patch. PIXL XRF scan area shown in black. White regions delineate Fe-Si material, which corresponds to white points in Figure 7. Images were cropped to remove abrasion fines and vignetting.

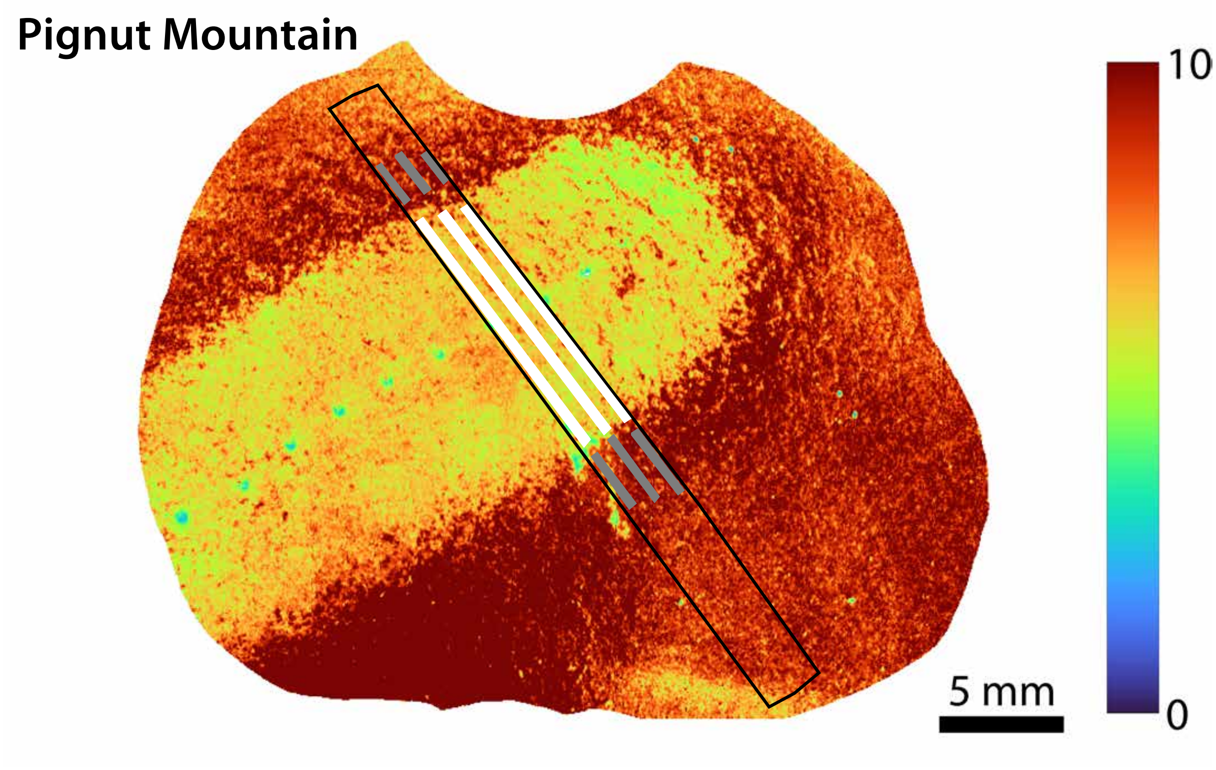

Figure S14: Map of NIR/UV ratio, as measured by PIXL's MCC, for the Pignut Mountain target, for which PIXL data were acquired on sol 463. After the SuperCam LIBS instrument acquired spot analyses on the target, clearing natural dust in the process, PIXL MCC and XRF data were acquired. PIXL XRF scan area, comprised of three adjacent line scans, shown in black. White regions delineate low-dust underlying rock, and grey regions delineate dust-rich natural surface. Images were cropped to remove vignetting.

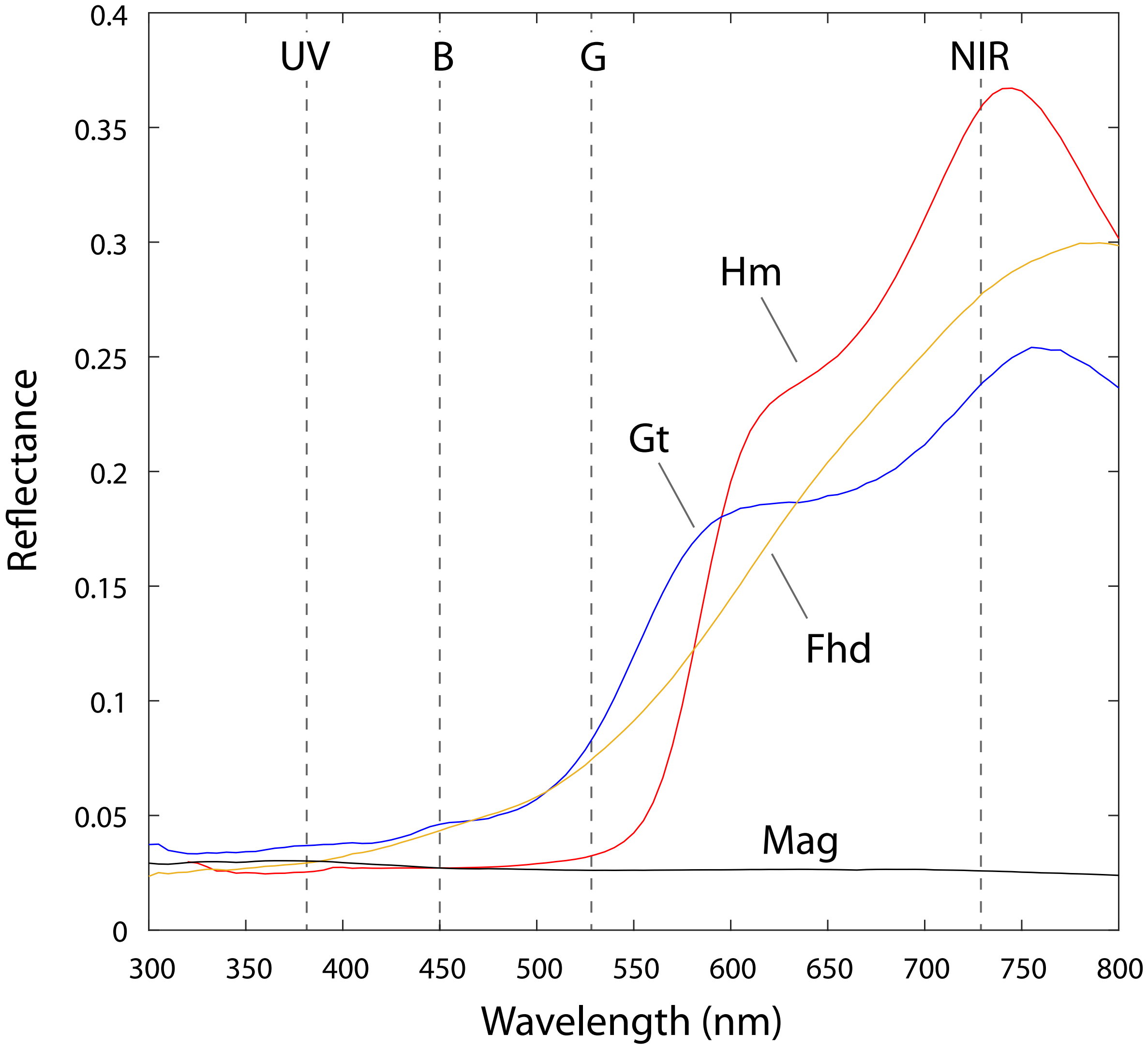

Figure S15: Reflectance spectra of major Fe-oxide minerals. All spectra were obtained from the RELAB spectral library and acquired via bidirectional diffuse reflectance spectroscopy. Measurement IDs are: hematite (synthetic; <140 nm; cahe01); goethite (synthetic; <145 μm; clgo01); ferrihydrite (synthetic; cljb45); magnetite (<45 μm; camg03).

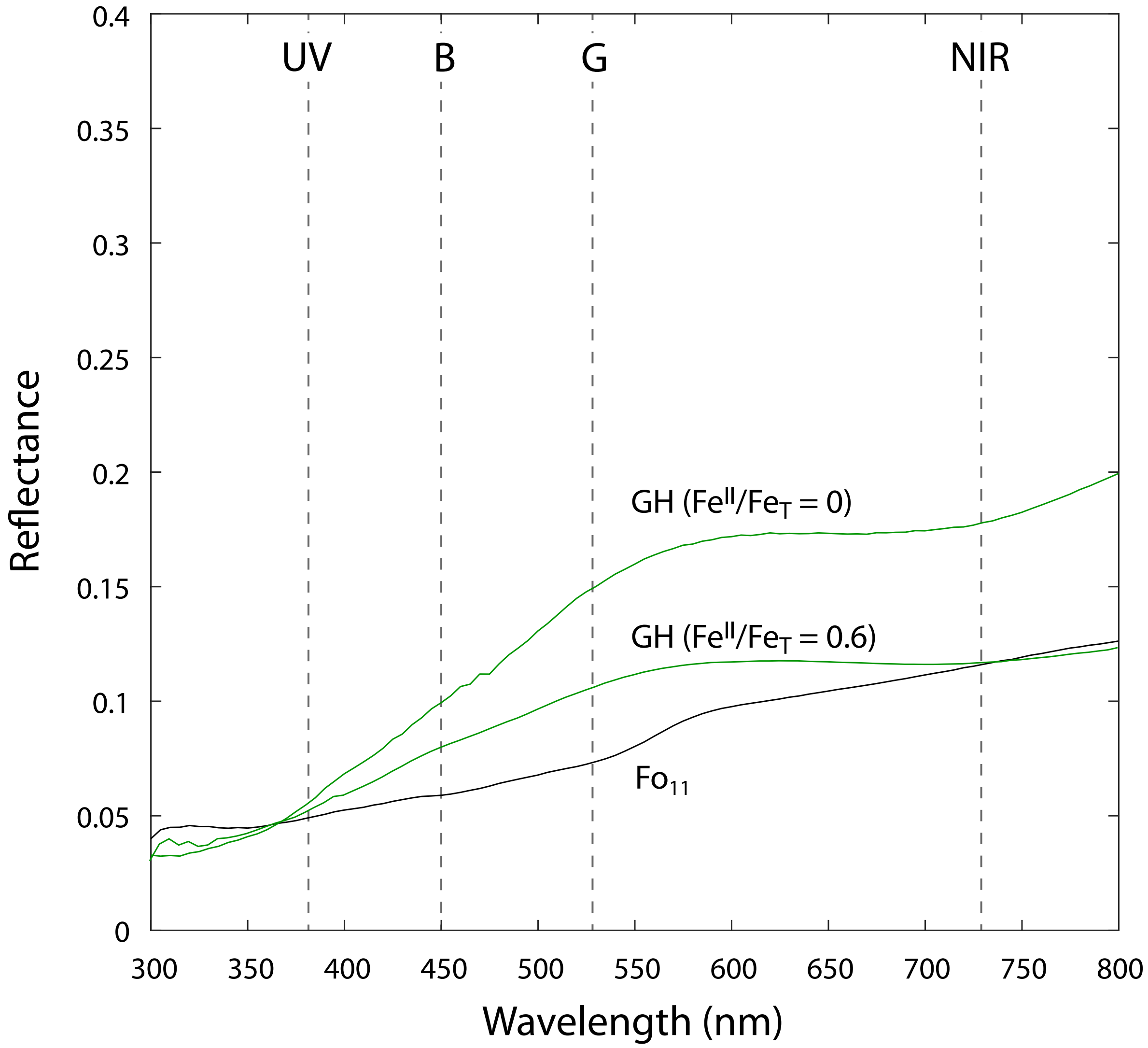

Figure S16: Reflectance spectra of major Fe-oxide minerals. All spectra were obtained from the RELAB spectral library and acquired via bidirectional diffuse reflectance spectroscopy. Measurement IDs are: greenalite-hisingerite Fe(II)/Fe(Total) = 0 ("hisingerite" from Gillinge, Sweden, sample number 48-1817 in (*71*); <45 μm; clrm138); greenalite-hisingerite Fe(II)/Fe(Total) = 0.6 ("greenalite" from La Union, Murcia, Spain; <45 μm; c1gr11a); olivine (Fo_{11}) (synthetic; cldd46).

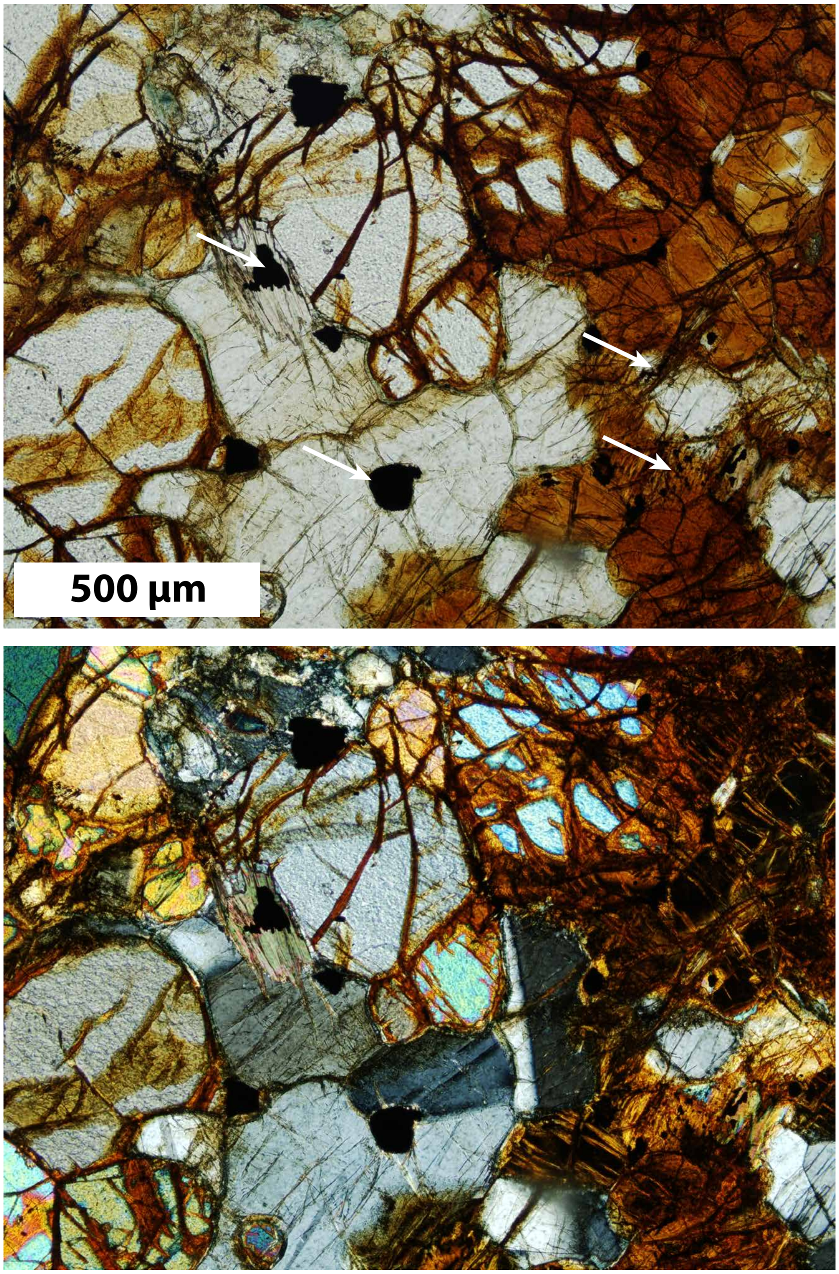

Figure S17: Plane polarised light (PPL; top) and cross polarised light (XPL; bottom) images of Duluth Complex sample BP-1. greenalite/hisingerite-rich areas (brown in PPL; moderately birefringent in XPL) are hosted in microfractures and larger fracture networks, and progressively replace olivine toward the right side of the image. Magnetite is present as finely crystalline opaque minerals hosted in nearly all fractures (indicated with arrows) and in some regions coalesce to form larger magnetite-rich accumulations.

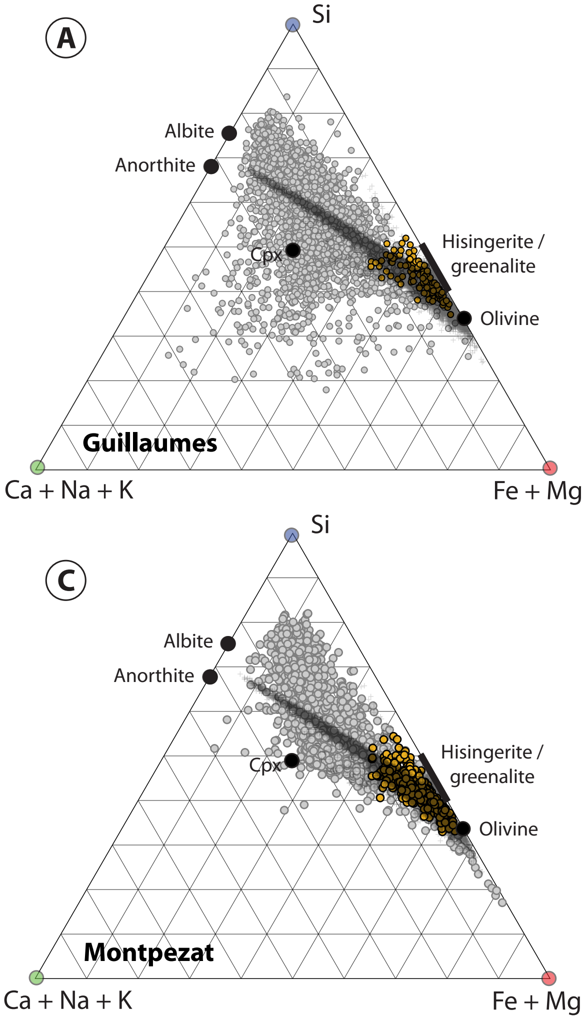

Figure S18: Comparison between PIXL XRF data from four Máaz formation abraded targets and laboratory micro-XRF measurements of Duluth Complex serpentinite (sample BP-1). Orange circles: individual PIXL XRF spot analyses corresponding to Fe-Si material; grey circles: individual PIXL XRF spot analyses corresponding to remaining portion of the PIXL scan; black circles: individual PIXL XRF spot analyses corresponding to high-Fe material within the Alfalfa target. Small grey crosses: individual laboratory XRF spot analyses of sample BP-1. All XRF data were corrected for surface roughness and diffraction effects as discussed in *Materials and Methods*.

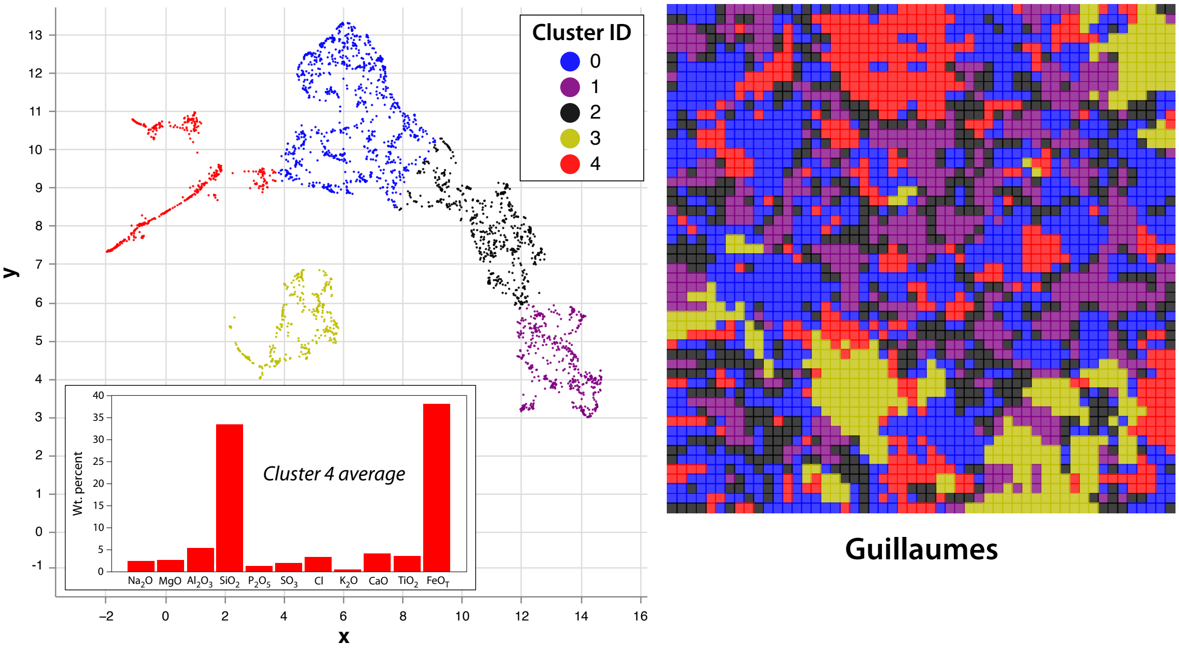

Figure S19: Visualisation of latent space (left) clustered using Gaussian mixture modeling applied to the Guillaumes PIXL XRF scan (*Materials and Methods*). Each cluster is designated with a different color and is characterised by distinct chemical signals. Cluster 4 corresponds compositionally (inset) and spatially (right) to the Fe-Si material identified through manual analysis. This analysis shows that cluster 4 represents an end-member that is chemically distinct from the remainder of the scan data. Spatial distribution of points within a cluster shown on the right of the figure disregards spatial coordinate data obtained during the scan and are plotted in numbered order of data acquisition.

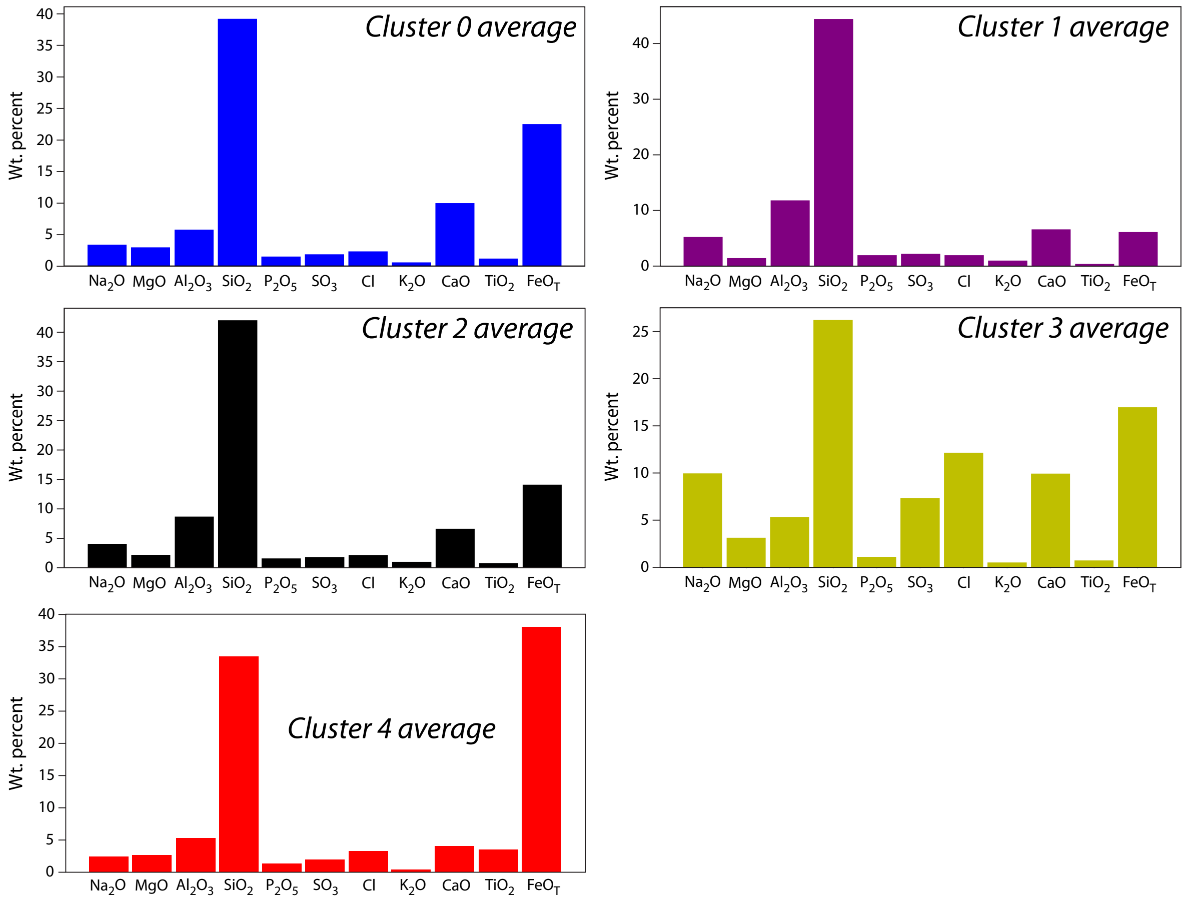

Figure S20: Average elemental composition of the clusters shown in Figure S19, normalised to 100%.

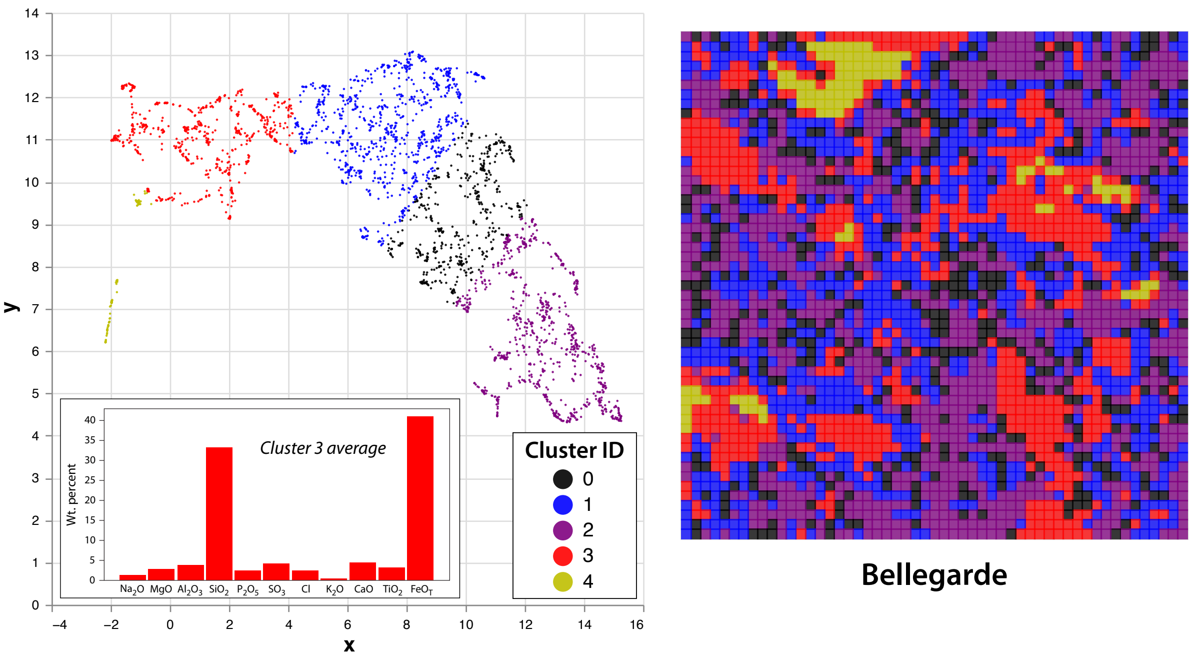

Figure S21: Visualisation of latent space (left) clustered using Gaussian mixture modeling applied to the Bellegarde PIXL XRF scan (*Materials and Methods*). Each cluster is designated with a different color and is characterised by distinct chemical signals. Cluster 3 corresponds compositionally (inset) and spatially (right) to the Fe-Si material identified through manual analysis. This analysis shows that cluster 3 represents an end-member that is chemically distinct from the remainder of the scan data. Spatial distribution of points within a cluster shown on the right of the figure disregards spatial coordinate data obtained during the scan and are plotted in numbered order of data acquisition.

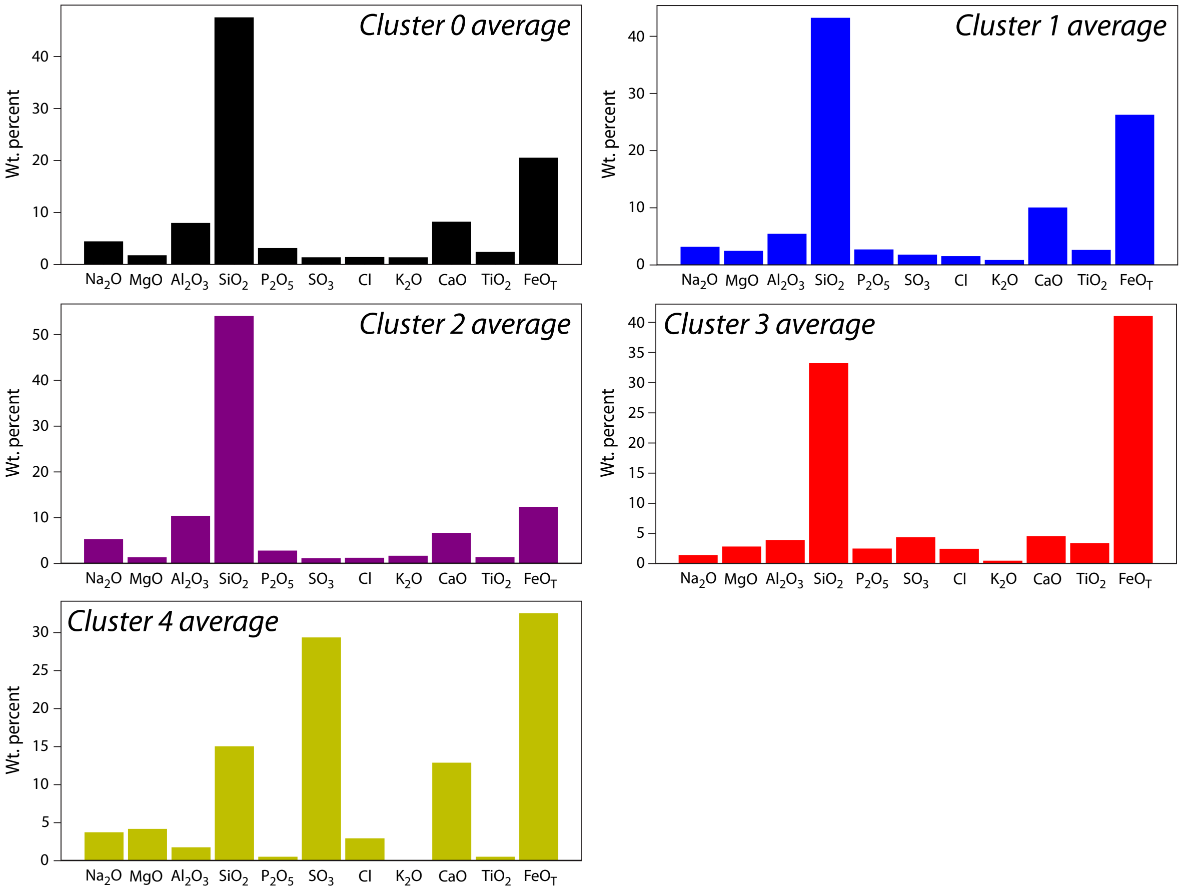

Figure S22: Average elemental composition of the clusters shown in Figure S21, normalised to 100%.

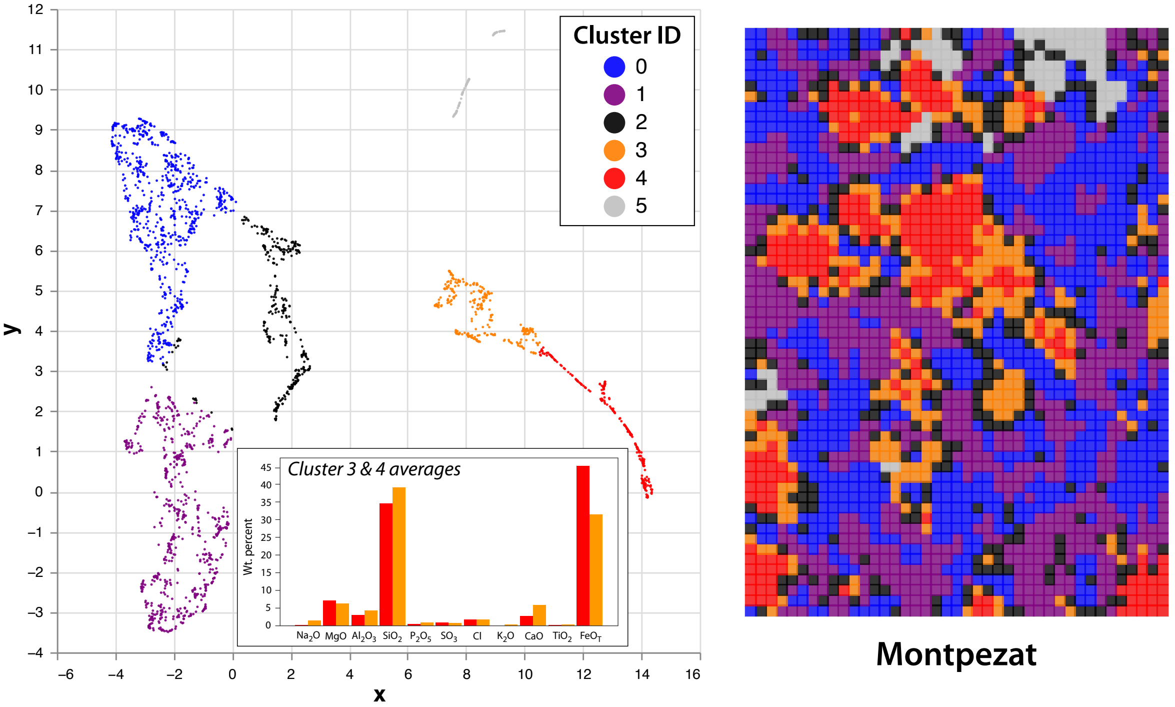

Figure S23: Visualisation of latent space (left) clustered using Gaussian mixture modeling applied to the Montpezat PIXL XRF scan (*Materials and Methods*). Each cluster is designated with a different color and is characterised by distinct chemical signals. Clusters 3 and 4 correspond compositionally (inset) and spatially (right) to the Fe-Si material identified through manual analysis. This analysis shows that clusters 3 and 4 together represent an end-member that is chemically distinct from the remainder of the scan data. Spatial distribution of points within a cluster shown on the right of the figure disregards spatial coordinate data obtained during the scan and are plotted in numbered order of data acquisition.

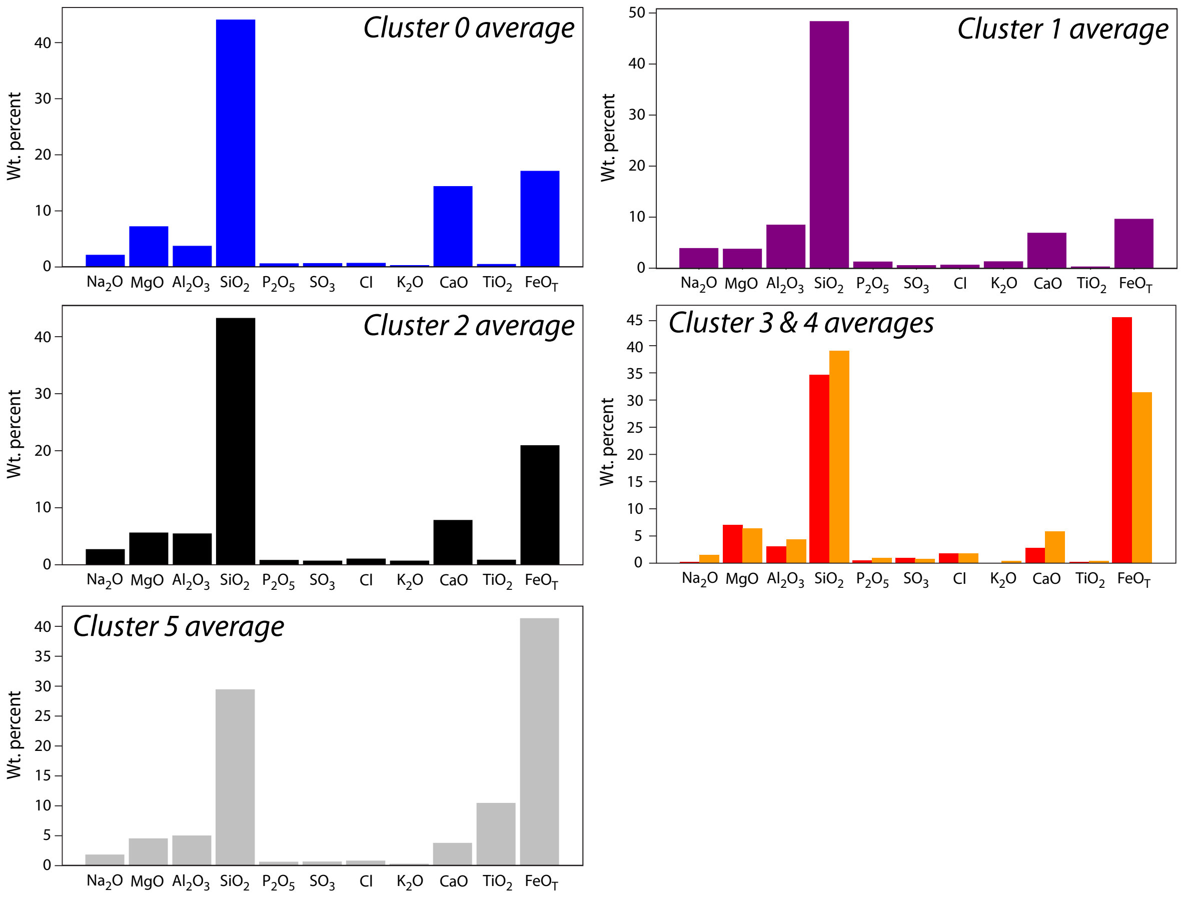

Figure S24: Average elemental composition of the clusters shown in Figure S23, normalised to 100%.

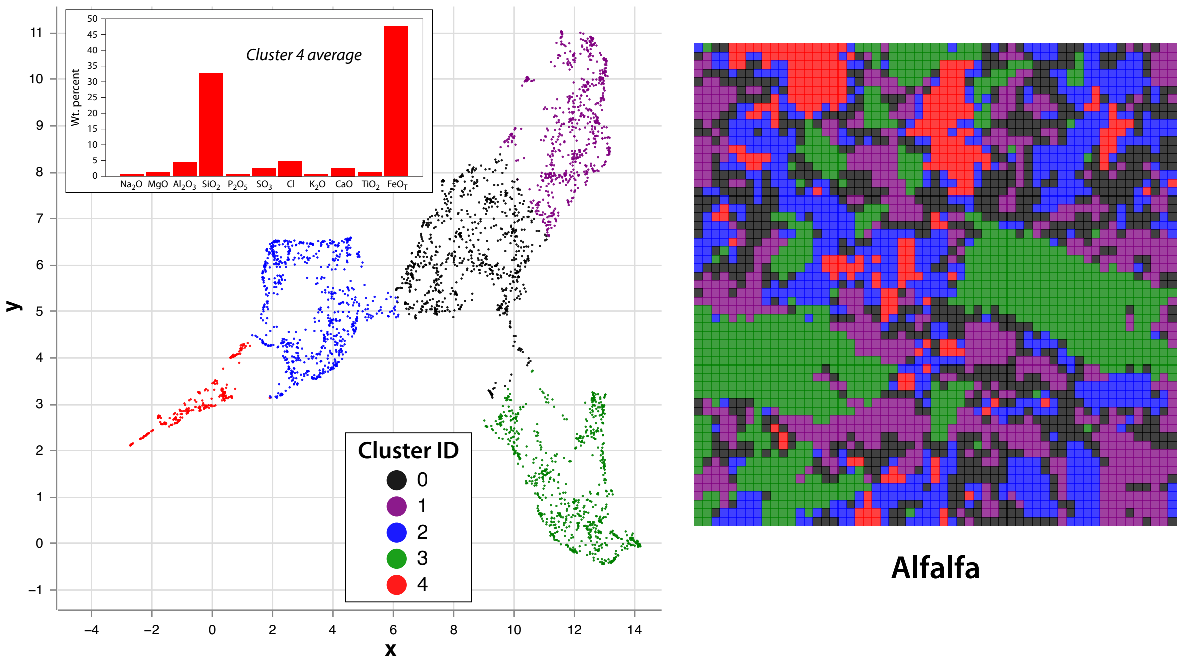

Figure S25: Visualisation of latent space (left) clustered using Gaussian mixture modeling applied to the Alfalfa PIXL XRF scan (*Materials and Methods*). Each cluster is designated with a different color and is characterised by distinct chemical signals. Cluster 4 corresponds compositionally (inset) and spatially (right) to the Fe-Si material identified through manual analysis. This analysis shows that cluster 4 represents an end-member that is chemically distinct from the remainder of the scan data. Spatial distribution of points within a cluster shown on the right of the figure disregards spatial coordinate data obtained during the scan and are plotted in numbered order of data acquisition.

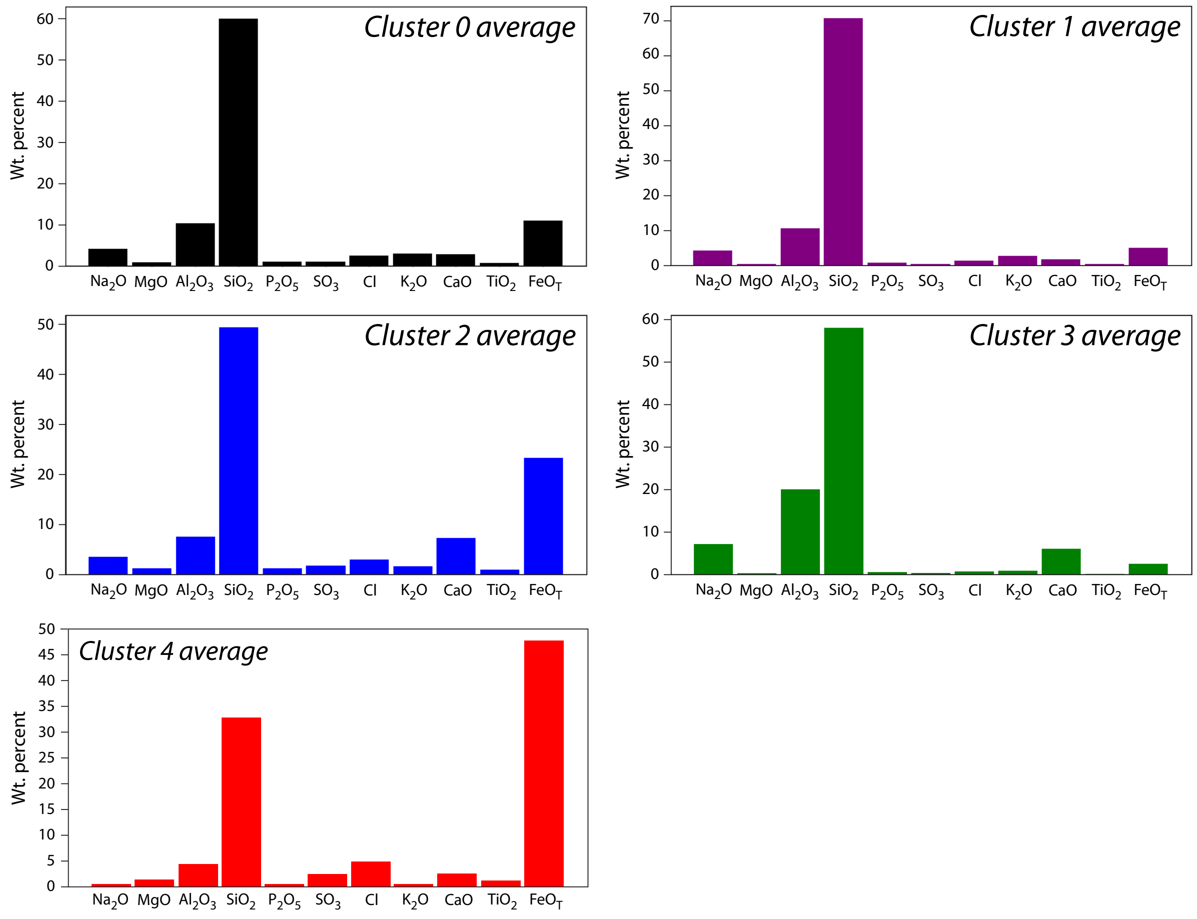

Figure S26: Average elemental composition of the clusters shown in Figure S25, normalised to 100%.

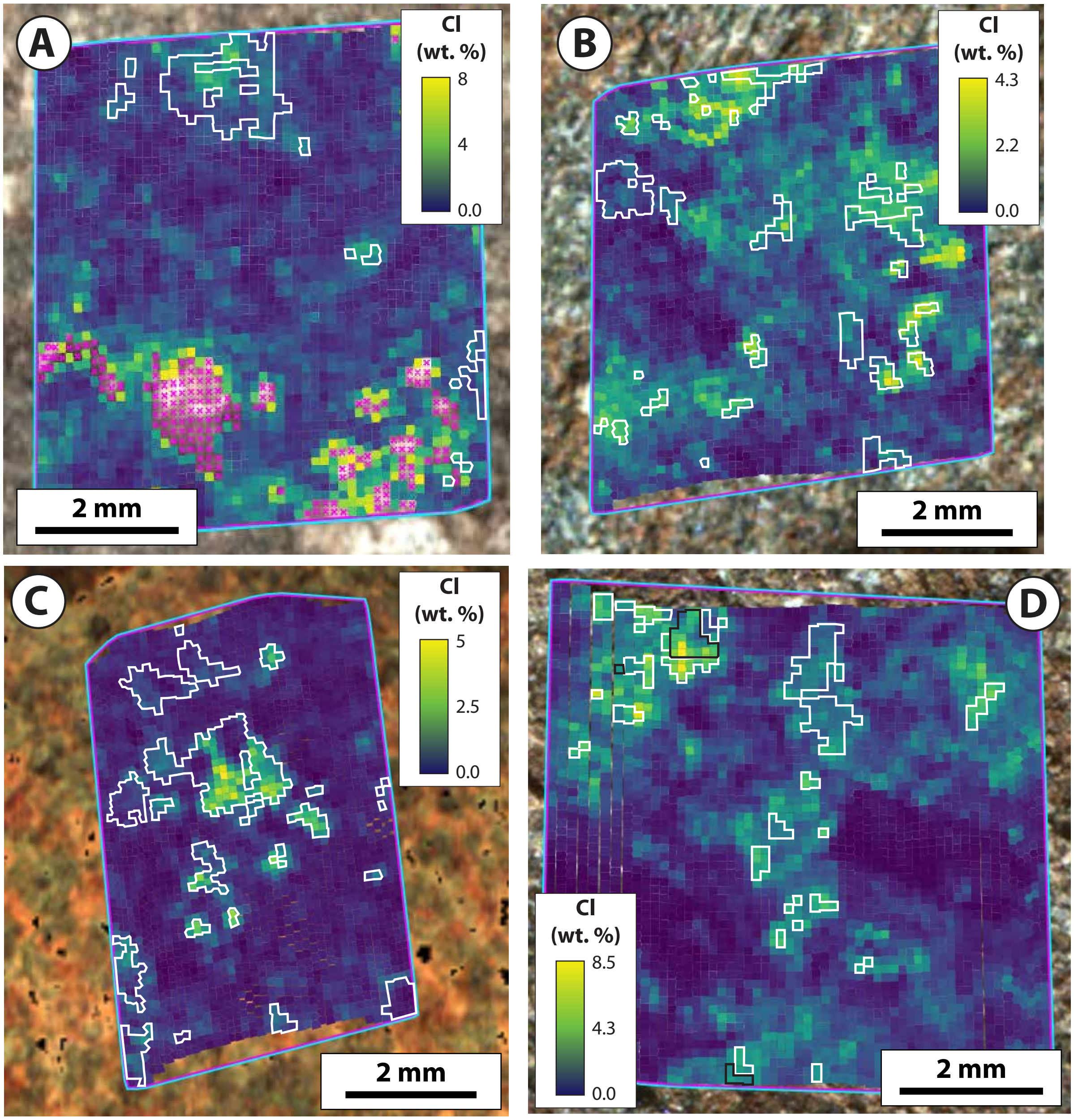

Figure S27: Cl abundance maps for the four Máaz formation abraded targets: Guillaumes (A), Bellegarde (B), Montpezat (C), and Alfalfa (D). White regions delineate Fe-Si material, which corresponds to orange points in Figure S28. All XRF data were corrected for surface roughness and diffraction effects as discussed in *Materials and Methods*.

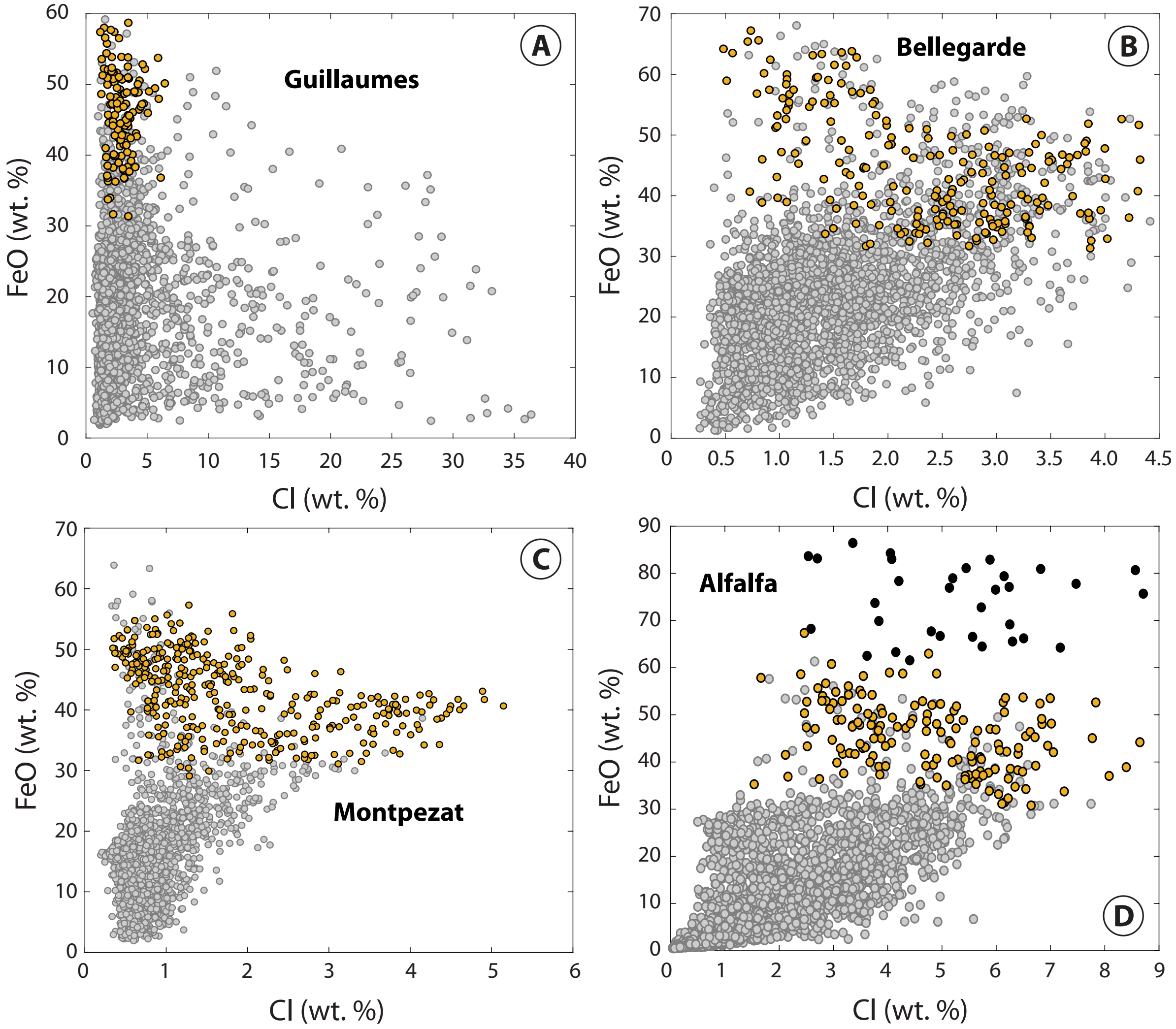

Figure S28: FeO versus Cl concentrations for the four Máaz formation abraded targets. Orange circles: individual XRF spot analyses corresponding to Fe-Si material; grey circles: individual XRF spot analyses corresponding to remaining portion of the PIXL scan; black circles: individual XRF spot analyses corresponding to high-Fe material within the Alfalfa target. All XRF data were corrected for surface roughness and diffraction effects as discussed in *Materials and Methods*.

Supplementary Tables

	Guillaumes Fe-Si	Bellegarde Fe-Si	Montpezat Fe-Si	Alfalfa Fe-Si	Alfalfa high-Fe
**Na_2O**	0.4	0.6	0.2	0.2	0.0
**MgO**	2.4	2.6	6.3	1.4	1.2
**Al_2O_3**	4.6	3.4	3.2	3.8	2.0
**SiO_2**	34.2	33.6	35.1	33.2	9.2
**P_2O_5**	0.9	2.1	0.4	0.3	0.0
**SO_3**	1.0	3.6	0.8	2.5	1.5
**Cl**	3.1	2.6	1.9	4.7	5.1
**K_2O**	0.2	0.3	0.1	0.3	0.0
**CaO**	2.3	3.7	3.1	2.4	1.5
**TiO_2**	0.1	0.8	0.1	0.1	0.0
**MnO**	0.8	0.6	0.7	0.9	1.6
**FeO_T**	46.3	43.4	42.2	48.5	77.0
**Sum**	96.5	97.2	94.1	98.3	99.1

Table S1: PIXL XRF bulk sum compositions for Fe-Si population and high-Fe population in Máaz formation abraded targets. Bulk sums were determined using the lower of the concentration of each element determined by sum spectra from X-ray detector A or B to limit the effects of X-ray diffraction. All values are in wt. %.

	Pignut Mountain (Dust-rich)	Pignut Mountain (Dust-poor)
**Na_2O**	2.6	1.8
**MgO**	8.6	12.4
**Al_2O_3**	7.8	7.0
**SiO_2**	36.0	35.7
**P_2O_5**	0.9	0.9
**SO_3**	11.5	15.5
**Cl**	1.6	1.7
**K_2O**	0.3	0.2
**CaO**	3.0	2.0
**TiO_2**	0.7	0.7
**MnO**	0.2	0.1
**FeO_T**	16.3	16.1
**Sum**	89.4	94.0

Table S2: PIXL XRF bulk sum compositions for dust-rich and dust-poor regions of the target Pignut Mountain. Bulk sums were determined using the lower of the concentration of each element determined by sum spectra from X-ray detector A or B to limit the effects of X-ray diffraction. All values are in wt. %.
